# Elucidation of genome-wide understudied proteins targeted by PROTAC-induced degradation using interpretable machine learning

**DOI:** 10.1371/journal.pcbi.1010974

**Published:** 2023-08-17

**Authors:** Li Xie, Lei Xie

**Affiliations:** 1 Department of Computer Science, Hunter College, The City University of New York, New York City, New York, United States of America; 2 Ph.D. Program in Computer Science, The Graduate Center, The City University of New York, New York City, New York, United States of America; 3 Helen and Robert Appel Alzheimer’s Disease Research Institute, Feil Family Brain & Mind Research Institute, Weill Cornell Medicine, Cornell University, New York City, New York, United States of America; Queen’s University, CANADA

## Abstract

Proteolysis-targeting chimeras (PROTACs) are hetero-bifunctional molecules that induce the degradation of target proteins by recruiting an E3 ligase. PROTACs have the potential to inactivate disease-related genes that are considered undruggable by small molecules, making them a promising therapy for the treatment of incurable diseases. However, only a few hundred proteins have been experimentally tested for their amenability to PROTACs, and it remains unclear which other proteins in the entire human genome can be targeted by PROTACs. In this study, we have developed PrePROTAC, an interpretable machine learning model based on a transformer-based protein sequence descriptor and random forest classification. PrePROTAC predicts genome-wide targets that can be degraded by CRBN, one of the E3 ligases. In the benchmark studies, PrePROTAC achieved a ROC-AUC of 0.81, an average precision of 0.84, and over 40% sensitivity at a false positive rate of 0.05. When evaluated by an external test set which comprised proteins from different structural folds than those in the training set, the performance of PrePROTAC did not drop significantly, indicating its generalizability. Furthermore, we developed an embedding SHapley Additive exPlanations (eSHAP) method, which extends conventional SHAP analysis for original features to an embedding space through in silico mutagenesis. This method allowed us to identify key residues in the protein structure that play critical roles in PROTAC activity. The identified key residues were consistent with existing knowledge. Using PrePROTAC, we identified over 600 novel understudied proteins that are potentially degradable by CRBN and proposed PROTAC compounds for three novel drug targets associated with Alzheimer’s disease.

## Author summary

Many human diseases remain incurable because disease-causing genes cannot by selectively and effectively targeted by small molecules. Proteolysis-targeting chimera (PROTAC), an organic compound that binds to both a target and a degradation-mediating E3 ligase, has emerged as a promising approach to selectively target disease-driving genes that are not druggable by small molecules. However, not all of proteins can be accommodated by E3 ligases, and be effectively degraded. Knowledge about the degradability of a protein will be crucial for PROTAC design. However, only hundreds of proteins have been experimentally tested if they are amenable to the PROTACs. This leaves us uncertain about which other proteins in the entire human genome can be targeted by PROTACs. In this paper, we propose an intepretable machine learning model, PrePROTAC, which takes advantage of powerful protein language modeling. PrePROTAC achieves high accuracy when evaluated with an external dataset which comes from different gene families from the proteins in the training data, suggesting the generalizability of this model. We apply PrePROTAC to the human genome, and identify more than 600 understudied proteins that are potentially responsive to PROTACs. Furthermore, we design PROTAC compounds for three novel drug targets associated with Alzheimer’s disease.

## Introduction

Despite rapid progress in the development of small-molecule drugs, traditional drug discovery is limited by the requirement for efficient inhibitions of functional binding sites in protein targets. However, many of disease-associated proteins neither have suitable binding pockets for small molecule drugs nor bind inhibitors with considerable affinity [1]. It is estimated that only 2–5% of the human genome is druggable by small molecule drugs [1, 2]. For example, in anti-cancer drug discovery, major cancer drug targets belong to serine/threonine/tyrosine kinases, growth factor receptors and GPCRs. Other classes of cancer-relevant proteins, such as phosphatases, transcription factors, and RAS family members, are difficult to be inhibited by small molecules and are considered to be understudied proteins. Thus, it is necessary to seek alternative approaches to target these proteins. Several types of biological drugs, including peptides, antibodies, modified nucleic acids, and vaccines, have been developed to target these proteins. However, the larger size of the biological drugs limits their delivery mode and makes it more challenging to alter intracellular targets compared to small molecule drugs. [3].

Different from small molecule and biological drugs, PROTAC molecules are hetero-bifunctional molecules that consist of a small molecule binding moiety for a target protein (i.e., warhead) and an E3 ubiquitin ligase-recruiting moiety coupled by a chemical linker. With the two binding moieties, PROTACs recruit an E3 ubiquitin ligase to a targeted protein and induce the degradation of the entire targeted proteins in the ubiquitin–proteasome system [4–6]. Therefore, even when the binding site of a PROTAC is not located on the functional domain for certain targeted proteins, such as undruggable proteins, PROTACs can disrupt their functions by facilitating the degradation of the entire protein [7–10]. This unique feature of PROTACs allows them to overcome the limitations of small molecule drugs when targeting undruggable proteins, including transcription factors, scaffolding proteins, and regulatory proteins [11–13].

PROTACs can enhance both selectivity and efficacy of targeted therapies. In the PROTAC-induced degradation, a ternary complex structure is formed by a targeted protein, a PROTAC, and an E3 ligase. The selectivity of PROTACs is determined not only by the warhead but also by the non-native protein-protein interactions (PPIs) between the E3 ligase and the targeted protein, as well as the stability of the ternary complex structure [5, 12, 13]. Even with a promiscuous warhead, PROTACs can still exhibit enhanced target selectivity compared to small molecule inhibitors [14]. Furthermore, low-affinity ligands or ligands that fail to modulate targeted functions are typically considered as ineffective ligands in the traditional drug discovery. However, they can still be utilized in the design of PROTACs to induce the degradation of target proteins [15].

Moreover, for multi-functional proteins, small molecule or biological drugs can only inhibit the occupied functional sites, whereas PROTACs can target all functions by eliminating the entire proteins. Thus, PROTACs can enhance pharmaceutical efficiencies. For instance, a PROTAC designed for receptor tyrosine kinases has been shown to inhibit both cell proliferation and downstream signaling [16]; and a focal adhesion kinase PROTAC has affected both tumor invasion and migration [17, 18]. Recently, the PROTAC-induced degradation has been applied to multi-protein complexes to modulate multiple functions on the entire complex through the degradation on one component, leading to destabilization and subsequent degradation of other complex components [19]. Such ability significantly expands the current drug target space and facilitates the addressing of drug resistance problems by targeting multi-component complex systems. There have been several successful cases in the degradation of epigenetic proteins, including epigenetic reader protein BRD4 [20–22], epigenetic eraser proteins [23] and others [15, 24, 25]. These studies have resulted in inhibitions of both enzymatic and scaffolding functions for epigenetic protein complexes. Studies on polycomb repressive complex 2 (PRC2) [26–28] have also demonstrated that PROTAC targeting of EED can lead to the loss of EED, EZH2, and SUZ12 proteins, disrupting histone methylation and suppressing cell proliferation.

Another advantage of PROTAC induced degradation over traditional inhibition is the ability to address acquired drug resistances [16, 29–31]. For instance, the PROTAC derived from the BTK inhibitor ibrutinib can induce degradation of both wild-type and C481S-mutant BTK, thereby overcoming ibrutinib resistance [30]. This study suggested that PROTACs can circumvent resistance mechanisms that affect parent inhibitors. Several studies have demonstrated that PROTACs can overcome drug resistance to the original targets by targeting other proteins. For example, a receptor tyrosine kinase (RTK) inhibitor lapatinib conjugated with a VHL ligand produced a PROTAC capable of inducing the degradation of both membrane-bound wild-type EGFR and disease-relevant EGFR mutants [16].

These intrinsic advantages of PROTACs’ mechanism of action make PROTAC-induced degradation a highly promising therapeutic modality. It has the potential to reduce drug exposure requirements, enhance drug selectivity, circumvent drug resistance mechanism, and target the undruggable disease genes. A growing body of research is focused on this field, encompassing both experimental and computational approaches. However, computer-based drug design for PROTACs is still limited to a small number of published ternary complex structures, and mainly involves the modeling of ternary complex structures with a pre-designed PROTAC through protein docking [32–34], combining with molecular dynamic simulations [35–38] and molecular modeling on linkers [5, 39]. Recently, Fischer et al. identified a group of degradable protein kinases and kinase-like proteins through systematically designed PROTAC-induced degradation experiments across the human kinome and developed a large library of protein kinase-targeting degraders [40]. Additionally, Hou et al. developed a web-based database called PROTAC-DB, which collects PROTAC related data through literature search and data processing [41]. These databases provide valuable information for computer-based PROTAC design.

However, no study has been conducted to explore understudied proteins in the entire human genome that could be amenable to PROTAC-induced degradation. In this work, we have developed an interpretable machine learning model called PrePROTAC. It utilizes embedding features extracted from protein sequences to predict degradable proteins targeted by PROTACs. PrePROTAC is the first model of this kind, enabling us to identify understudied proteins whose degradability can be induced by PROTACs on a genome scale. We have identified approximately 615 new proteins responsible for multiple diseases that can be targeted by the PROTACs. Furthermore, we screened the PROTACs for three novel drug targets related to Alzheimer’s disease.

## Results and discussion

### Overview of PrePROTAC

**Fig 1:**
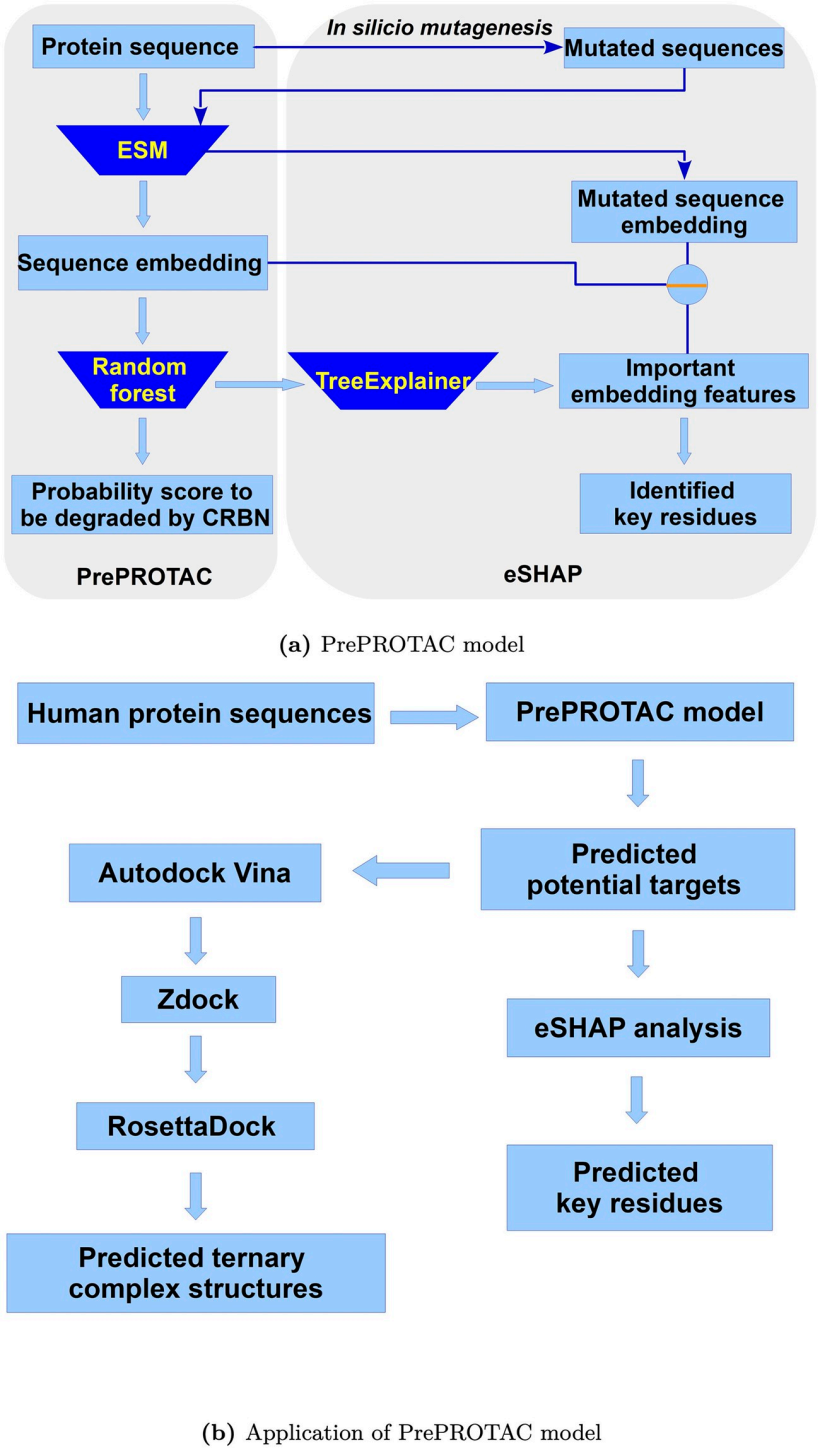
Overview of the PrePROTAC model and application process.

The workflow of our PrePROTAC model was shown in Fig 1a. Starting from a protein sequence, a sequence embedding was generated using protein language model ESM [42]. The sequence embedding was then used as features for a Random Forest (RF) classification model to predict the degradability of this protein targeted by CRBN. Meanwhile, an eSHAP interpretative model was developed to identify the key residues on the target protein by calculating the difference between the selected features of the original sequence and its single-residue mutated sequences. The details of the model training, validation and eSHAP analysis can be found in the Methods section.

The trained PrePROTAC model was applied to the whole human proteome, using a threshold of 0.9 to select proteins with the potential to be degraded by CRBN when induced by PROTACs. As shown in Fig 1b, Autodock Vina, Zdock and Rosseta docking were used to screen the PROTACs and build ternary complex structures for selected proteins. eSHAP analysis was used to identify the residues which might play important roles in the degradation.

### Comparison between Random Forest and Gradient Boost Tree classification methods with Ifeatures, D-script contact features and ESM features

Since there were only less than 500 proteins with annotated degradability information, but protein sequences were usually represented by high dimensional vectors, we selected Random Forest (RF) and Gradient Boost Tree (GBT) to train the prediction model. This choice was made because these algorithms are less prone to overfitting when working with high-dimensional data and a small number of samples. In order to achieve optimal performances, we tuned the hyper-parameters of the RF and GBT models with different features using 5-fold grid search cross-validation. The optimized hyper-parameters for different models and features were listed in Table A and B in S1 Text.

With the tuned hyper-parameters, RF and GBT classification models corresponding to 21 Ifeature descriptors [43], D-Script contact feature [44] and ESM feature [42] were trained to fit the protein kinase degradation data. The Repeated Stratified 5-Fold cross validation method was applied to validate these models. In this case, the Stratified 5-fold cross validation was repeated twice to get a more accurate estimate of the performance distribution of the model. The ROC-AUC scores for a total 42 classification models were shown in Fig A in S1 Text.

**Fig 2:**
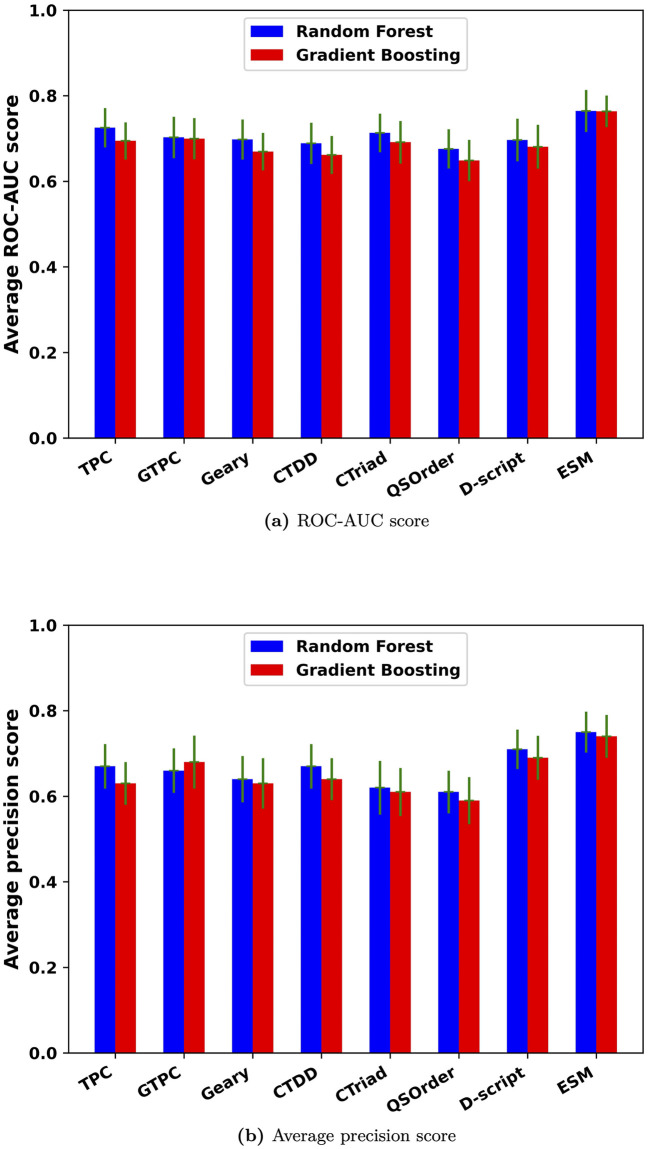
ROC-AUC scores and average precision scores for RF and GBT classification models with 8 features. (a) ROC-AUC score. Green line represents the standard deviation for ROC-AUC score. (b) Average precision score.

For the 21 Ifeature descriptors, the one with the best ROC-AUC score in each group was selected and compared with the D-script and ESM features. Models based on TPC, GTPC, Geary, CTDD, CTriad and QSOrder had the best performances compared with the other descriptors in the same groups. As shown in Fig 2, the RF model with the ESM feature demonstrated the best performance when evaluated by ROC-AUC scores and average precision (AP) scores. The GBT model with the ESM feature showed comparable performance to the RF model, with ROC-AUCs of 0.764 and 0.765, and precision scores of 0.740 and 0.750, respectively. Among TPC, GTPC, Geary, CTDD, CTriad, QSOrder, D-script contact feature and ESM feature, the ESM feature outperformed all others. Consequently, the ESM feature was exclusively used in the follow-up studies. The ROC curves, false positive rate curves, and precision-recall curves for the RF and GBT with TPC, GTPC, Geary, CTDD, CTriad, QSOrder, D-script contact feature and ESM feature can be found in Figs B-G in S1 Text.

The D-script contact feature, which is trained to predict native PPIs, may not be suitable for representing the formation of E3 ligase-PROTAC-target complex structures. Unlike native PPIs, E3 ligand and target proteins may not form a stable PPI complex without using RPOTAC as a molecule glue between them. Simply using native PPI predictions could not identify the CRBN-target pairs. When D-script was applied to predict interactions of 265 degradable proteins with CRBN in both training set and test set, none of them could be identified to interact with CRBN. Another PPI prediction method, PrePPI [45] [46], was also used to predict the interactions between these degradable proteins and CRBN. Only 10 out of the 265 proteins were identified based on the complex structures between the target proteins and CRBN.

In order to investigate whether the performance could be improved by adding other features in combination with the ESM feature, TPC, GTPC, Geary, CTDD, CTriad, QSOrder and Dscript features were combined with the ESM feature. Hyper-parameters for the RF models with these combined features were trained and 5-fold cross validations of these models were performed on the training set. The ROC-AUC scores and AP scores were shown in Fig H in S1 Text. Even when compared with these combined features, the performances of the ESM feature alone remained the best. Simply adding these features to the ESM feature could not improve the performance significantly.

We also investigated whether the performance could be improved when combining the RF and GBT models. Two different ensemble methods were used: the soft voting method and the consensus method. In the soft voting method, the average probability scores of the RF and GBT models were used to predict the degradation of proteins. For the consensus method, a positive result was only obtained when both models agreed, otherwise the result was negative. The performances of the soft voting model and consensus model were evaluated using the five-fold cross-validation method on the training set. The ROC-AUC, false positive rate-threshold, precision-recall curves for them were shown in Fig I in S1 Text. Both ensemble models showed similar performances to the RF model. When the ensemble models were evaluated using the external test set, their performances were better than the GBT model but still worse than the RF model, as shown in Fig J in S1 Text. In general, for all the tested models based on two different classification methods with 23 different features, the RF classification model with the ESM feature performed the best and would be used in the subsequent studies.

### Performance evaluation using external test data sets

We further rigorously evaluated the performance of models using an external test data set, where none of the proteins contain structural domains belonging to the SCOP protein kinase-like fold that was used in the training data (See Methods for details). The Structural Classification Of Proteins (SCOP) database [47, 48] manually classifies protein structures based on their structural similarities and evolutionary relationships into four levels: class, fold, superfamily, and family. Proteins in different SCOP folds usually do not share common structural features. In this test set, the positive samples include 50 proteins that could be degraded by CRBN through PROTAC-induced degradation. As shown in Fig K in S1 Text, none of proteins in the test set has the e-value of sequence similarity less than 0.01 when compared to the proteins in the training set, as determined by BlastP [49]. This indicates that sequence features in the testing data are significantly different from those protein kinase sequences in the training set.

**Fig 3:**
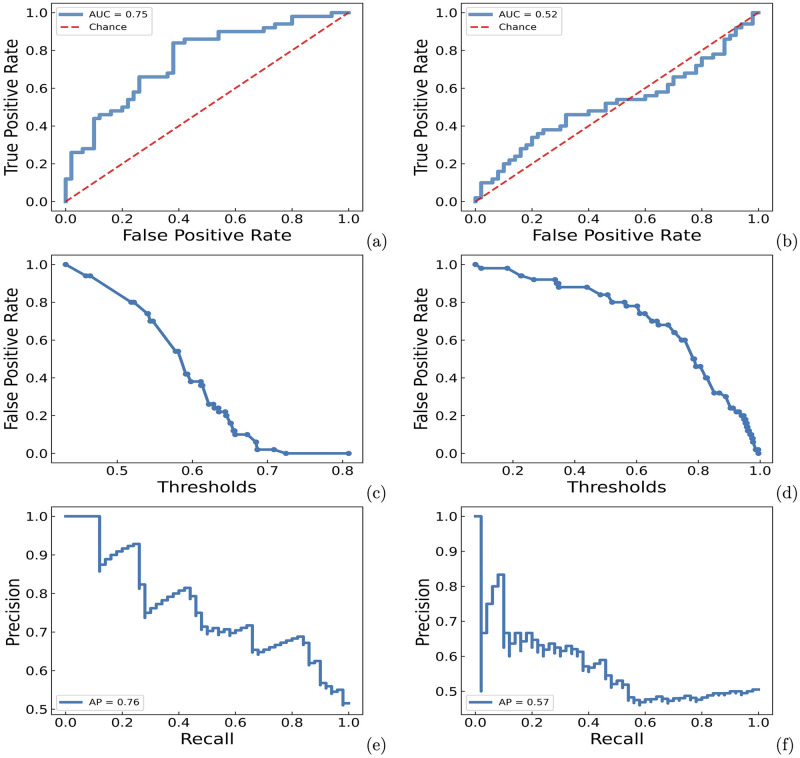
ROC-AUC, false positive rate-threshold, precision-recall curves for RF (left panel) and GBT (right panel) models on the external test set in which the negative samples are not in the sample folds with the positive samples. (a) ROC-AUC curve for the RF model. (b) ROC-AUC curve for the GBT model. (c) false positive rate-threshold curve for the RF model. (d) false positive rate-threshold curve for the GBT model. (e) precision-recall curve for the RF model. (f) precision-recall curve for the GBT model. AUC represents the average ROC-AUC score, AP represents the average precision score.

The negative samples were randomly selected proteins whose structures were not in the same fold as the positive targets, according to SCOP. The RF model achieved an ROC-AUC of 0.754±0.024 and an AP score of 0.760±0.026 for the prediction on this test set, as shown in Fig 3. These values were not significantly different from the ROC-AUC (0.760) and AP (0.750) obtained from the cross-validation experiments on the training set, indicating that the RF model did not suffer from significant overfitting. In comparison, the GBT model performed worse on the external test set, with an ROC-AUC of 0.518±0.035 and an AP score of 0.571±0.033.

We also included randomly sampled negative cases that were not the protein kinase but shared the same fold as one of structural domains in the positive multi-domain proteins. Because the structure of negative cases had certain similarity to that of positive cases, there was a possibility that a case labeled as negative could actually be positive. Consequently, the validation results might exhibit lower ROC-AUC and AP scores than it should be. As shown in Fig L in S1 Text, the ROC-AUC and AP score of the RF model slightly decreased to 0.721±0.026 and 0.681±0.035, respectively. Overall, the RF model is generalizable to predict the degradability of unseen proteins that are not protein kinases.

### eSHAP analysis identified key residues for the PROTAC activity of protein kinases

**Fig 4:**
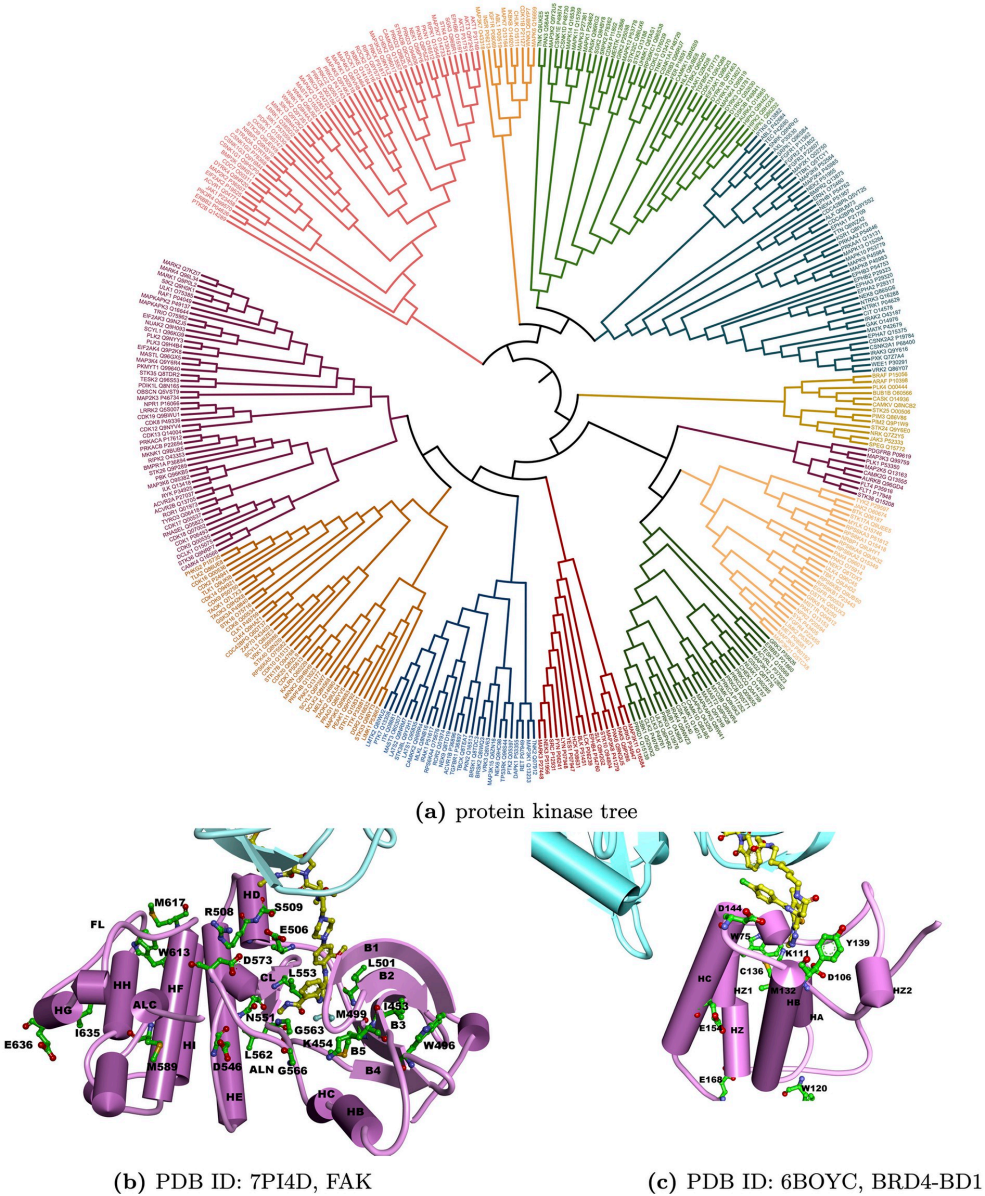
Protein kinase tree based on the key-position only MSA and structural mapping of the key positions for FAK and BRD4. In (b) and (c), cyan represents the structure of CRBN; purple represents the structure of FAK and BRD4; yellow balls and sticks represent PROTACs for FAK and BRD4; green balls and sticks represent the predicted key positions.

eSHAP analysis was applied to protein kinases in the training set to identify the key positions responsible for PROTAC activity. To gain further insights into the functional roles of these key positions, they were mapped to the structures of these protein kinases aligned across the protein kinase family. Structure-based multiple sequence alignments (MSAs) were obtained from 497 human protein kinase domains [50]. The identified key positions of the protein kinases were then mapped on the MSA. A key-position only MSA was generated by excluding the other residues, creating a focused MSA solely consisting of the key positions. The protein kinases were subsequently classified into 12 subgroups based on this key-position only MSA. For a better visualization of such classification, a protein kinase tree related to the key-position MSA was constructed using the maximum likelihood algorithm in the software MegaX [51]. The resulting tree was then visualized using the webserver iTOL [52]. As shown in Fig 4, different from typical protein kinase phylogenetic trees built from the MSA of whole protein kinase domains [50] [53] [54], the branches in this tree, generated from the key-position only MSA, contained protein kinases from different protein kinase subfamilies.

In these protein kinases, FAK has a ternary complex structure with CRBN and PROTAC. The key positions identified for FAK were mapped on the complex structure (PDB ID: 7PI4). As shown in Fig 4, most of these key positions were found in the PROTAC binding pocket or the interface between FAK and CRBN. These positions include ILE453 and LYS454 on the *β*-strand 3, TRP496, MET499 and LEU501 on the strand 5, GLU506, ARG508 and SER509 on the D helix, ASP546 and ASN551 on the catalytic loop, LEU553, LEU562 and GLY563 on the N terminal of the activation loop (ALN), GLY566 on the DFG motif (part of ALN), ASP573 and Met589 on the C terminal of the activation loop (ALC), TRP613 and MET617 on the F helix, and ILE635 and GLU636 on the G helix. All of these segments are of functional importance for the protein kinase.

LYS454 on the *β*-strand 3 is the catalytic residue that typically interacts with the *α*- and *β*- phosphates of ATP. MET499 is the gatekeeper residue that influences the accessibility to a buried region at the end of the ATP binding pocket and controls inhibitor sensitivity of protein kinases. Other residues on the *β*-strand 3 and 5 contribute to the formation of the buried region in the ATP binding pocket. ASP546 and ASN551 are located on the catalytic loop and are in close proximity to the gamma-phosphate group of ATP, making them directly involved in the catalytic functions of FAK. LEU553, LEU562 and GLY563 are located on the ALN, while GLY566 is one of the DFG residues. ALC contains the APE motif, which can stabilize the activation loop by docking to the F helix. MET589 is adjacent to the APE motif. GLY566 and ASP573 on the activation loop could bind to the substrates and promote catalysis. The F helix serves as a stable anchor for several other motifs in the C-lobe, including the catalytic loop and the activation loop, through hydrophobic contacts. The G helix is a solvent exposed helix and a part of the GHI-domain, which can interact with various substrate proteins and regulatory proteins, acting as allosteric sites. The key positions identified for other protein kinases also correspond to these functionally important segments. Representatives of the positive and negative samples for each protein kinase subgroup were selected based on the key-position only MSA. The structural mapping of their key positions and the key-position only MSA were shown in Figs M-X in S1 Text.

Besides FAK, the BD1 domain of BRD4 (PDB ID: 6BOY) also form a ternary complex structures with CRBN and its PROTAC. The key positions for this protein were also identified and mapped on their complex structures to elucidate the functional roles of these key positions. BRD4 is a member of the BET family, which regulates the expression of numerous immunity-associated genes and pathways. It is also a well-studied target protein for PROTAC-induced degradation [20, 34, 55]. The BD1 domain of BRD4 can be degraded by CRBN through PROTAC-induced degradation. The top five key positions on BD1 were shown on the ternary complex structures with CRBN and PROTAC. CYS136, TYR139 and ASP144 are the ligand binding residues, while the other top positions are also located near the ligand-binding pockets. In summary, the key residues identified by the eSHAP analysis align with existing biological knowledge, providing further evidence to support the prediction power of PrePROTAC.

### Application on human disease-associated understudied proteome

**Fig 5:**
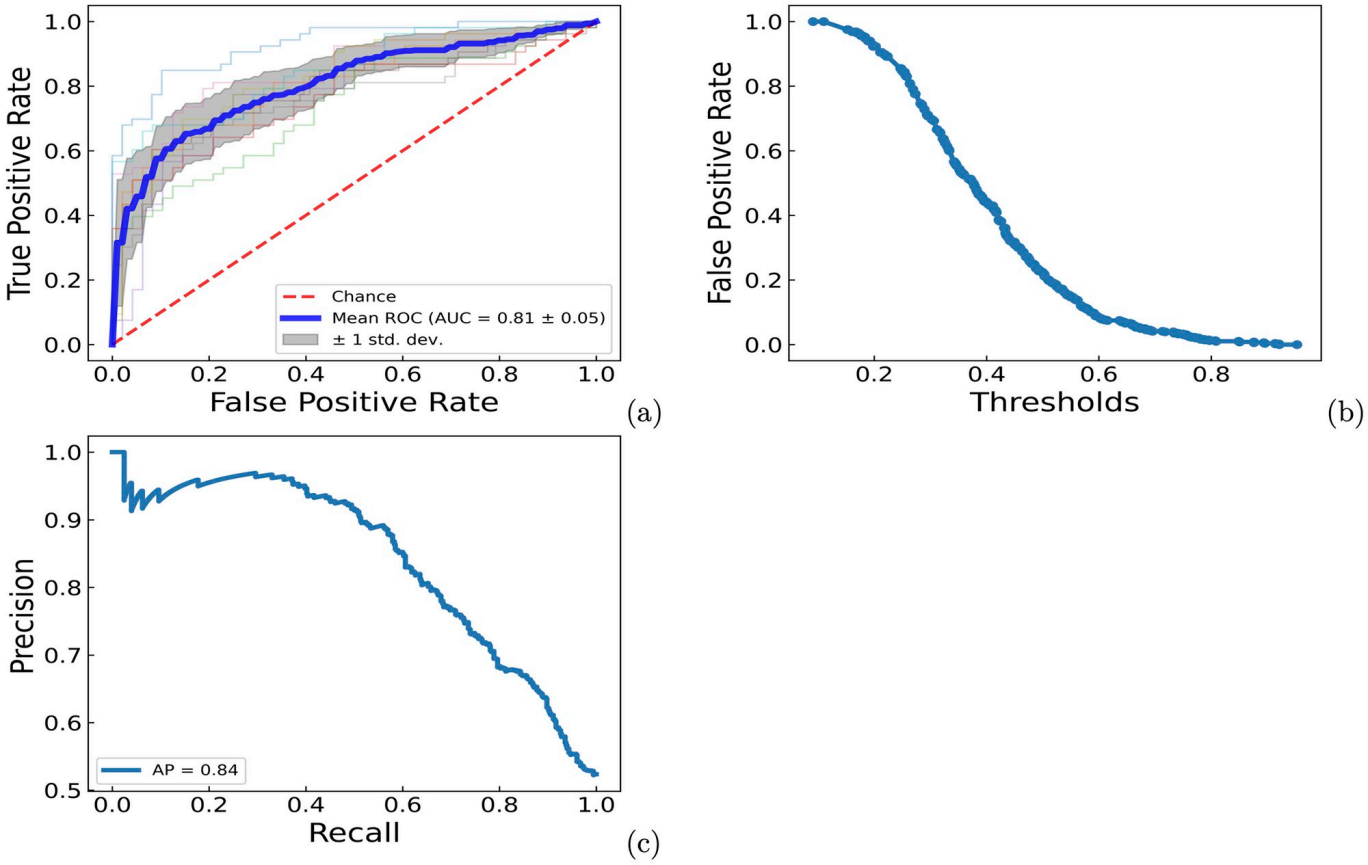
ROC-AUC curve, false positive rate-threshold curve and precision-recall curve for ten ESM RF models. a) ROC-AUC curve. b) False positive rate-threahold curve. c) Precision-recall curve.

To apply PrePROTAC to unseen proteins on a genome scale, we further developed a soft voting classification model based on the ten models with equal weight (See Methods for details). When the model was evaluated by the available data from all samples, ROC-AUC curves, false positive rate-threshold curve and precision-recall curve for the ten models were shown in Fig 5. Based on the correlation between the average false positive rate and the threshold from ten validation sets, setting the threshold to 0.85 resulted in a false positive rate of 0.004, indicating that only one of the negative samples was predicted as positive, considering there were 241 negative samples in the data set. In order to select potential degradable proteins with high confidence, the threshold of 0.90 would be used to identify positive hits in the prediction.

We applied PrePROTAC to 20,504 human proteins. The probability of these proteins being degraded by CRBN were predicted by the soft voting model and the distribution of probability scores were presented in Fig Y in S1 Text. Among them, there are 12,475 understudied disease associated human proteins (see Methods for selection procedure). Filtered by a probability score of 0.90, 615 of them were predicted to be degradable by CRBN with the help of PROTAC binding. None of them consist of the protein kinase-like domain. Information about these proteins and their predicted probability scores were listed in the supplementary Table C in S1 Text.

In order to design PROTACs for the proteins predicted positive by our PrePROTAC model, protein-protein docking and protein-ligand docking were employed to search for PROTACs which are able to bind both CRBN and selected target proteins. Three proteins related to Alzheimer’s diseases were chosen based on their PrePROTAC prediction scores. These proteins include Ski-like protein fragment (UniProt ID: P12757), death domain-associated protein 6 (UniProt ID: Q9UER7) and activating signal cointegrator 1 complex subunit 2 (UniProt ID: Q9H1I8). These proteins are highly expressed in the brain of Alzheimer’s disease patients and are associated with the risk of AD [56–59].

**Fig 6:**
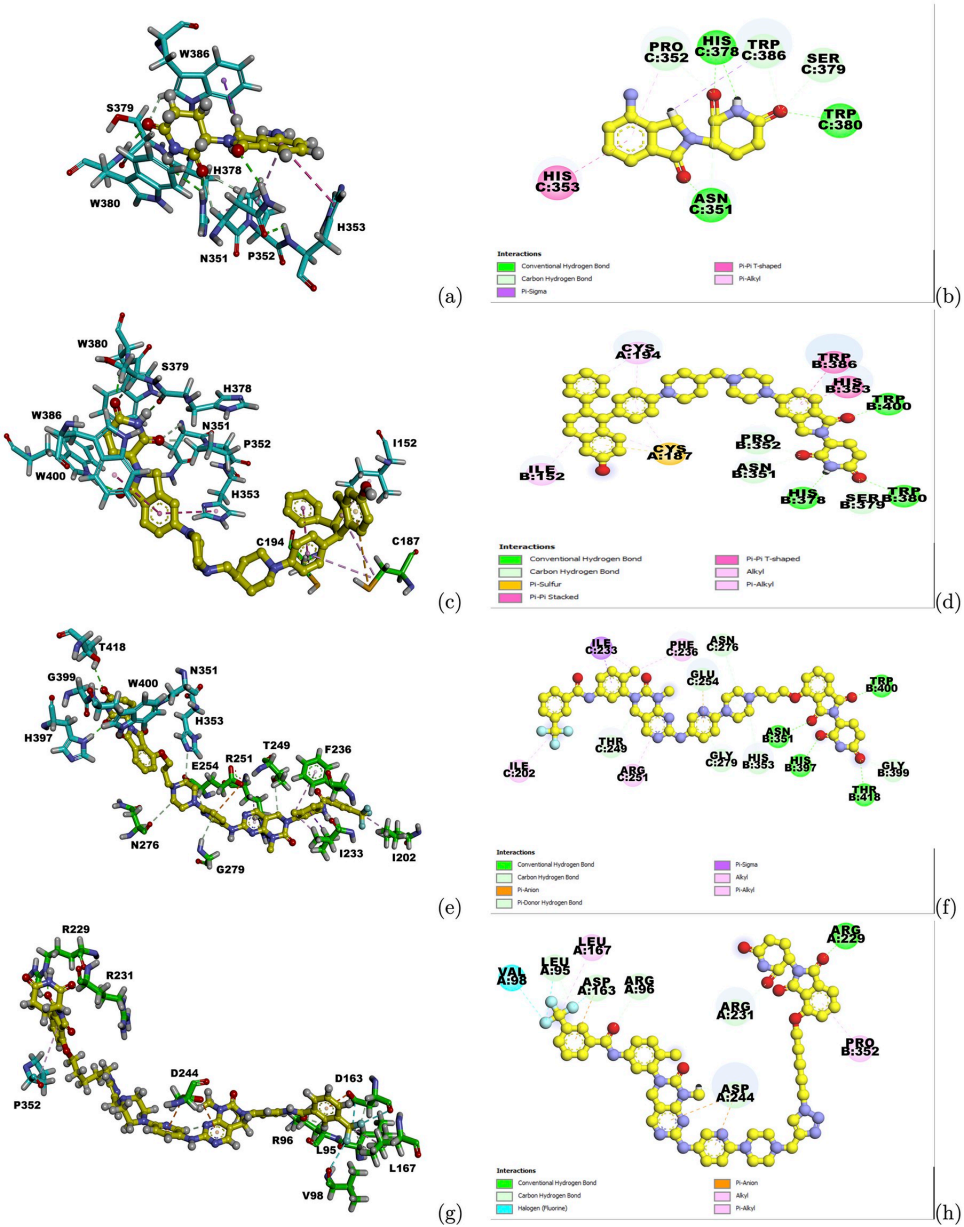
Interactions between predicted PROTACs and the CRBN-target protein complex structures. (a) Ligand-target Interactions between CRBN and its crystallized ligand LVY in 4TZ4. (b) 2D Ligand-target Interactions between CRBN and its crystallized ligand LVY in 4TZ4. C represents the PDB chain ID for CRBN. (c) Ligand-target interactions between PROTAC3269 and the complex structure of P12757 and CRBN. (d) 2D Ligand-target interactions between PROTAC3269 and the complex structure of P12757 and CRBN. A represents the chain ID for the target protein. B represents the chain ID for CRBN. (e) Ligand-target interactions between PROTAC3055 and the complex structure of Q9UER7 and CRBN. (f) 2D Ligand-target interactions between PROTAC3055 and the complex structure of Q9UER7 and CRBN. C represents the chain ID for the target protein. B represents the chain ID for CRBN. (g) Ligand-target interactions between PROTAC2145 and the complex structure of Q9H1I8 and CRBN. (h) 2D Ligand-target interactions between PROTAC2145 and the complex structure of Q9H1I8 and CRBN. A represents the chain ID for the target protein. B represents the chain ID for CRBN. Cyan and green sticks represent interacting residues on CRBN and the target protein, respectively. Yellow balls and sticks represent the ligand structures. Color representations for the interaction types are shown in figure legend.

Protein-protein docking methods Zdock and Rosetta were utilized to generate complex structures between CRBN and these target proteins. The starting structure for CRBN was obtained from the ternary complex structure of CRBN-PROTAC-BRD4 (PDB id: 6BOY) and the ligand mimic E3 ligase moiety was sourced from the CRBN-ligand complex structure (PDB id: 4TZ4, ligand: Lenalidomide (LVY)). Using the selected complex structure, docking was performed to screen three sets of compound libraries, including 2,074 CRBN related PROTACs in PROTAC-DB, 1,920 fragment-based compounds from high fidelity fragment library of Enamine [60] and 2,016 drugs provided by MedChem Express, LLC (Monmouth Junction, NJ, USA) in ZINC database [61]. The PROTACs with the best docking scores and conformations were selected for these target proteins after the compound screening on PROTAC-DB. In order to compare the predicted binding poses of the selected PROTACs with the interactions between CRBN and its co-crystallized ligand, 3D and 2D interactions between the predicted PROTACs and the CRBN-target protein complexes were visualized using Discovery Studio [62] and were shown in Fig 6.

Compound PROTAC3269 was selected for P12757 and its E3 ligase moiety precisely occupied the binding pocket of Lenalidomide in CRBN. All interactions between Lenalidomide and CRBN aere retained in the PROTAC3296, including interactions with ASN351, PRO352, HIS353, HIS378, SER379, TRP380 and TRP386. Similarly, for the other two target proteins, several such interactions were observed between CRBN and the CRBN ligand moiety on the predicted PROTACs. For instance, interactions between residues ASN351 and HIS353 and PROTAC3055 were maintained for Q9UER7, and the interaction between PRO352 and PROTAC2145 remained for Q9H1I8, resembling the interactions between CRBN and the co-crystallized ligand LVY. Other binding site residues on these target protein-CRBN complex structures were different from those in the original CRBN-ligand complex structure, but they still formed similar hydrogen bonds with oxygen atoms on the ring of the CRBN ligand moiety. On the warhead side, numerous residues on the target proteins also formed favorable interactions with the warheads on these PROTACs. These interactions between CRBN and E3 ligase ligand moieties, as well as between the target proteins and warheads, indicate the predicted PROTACs could form dual bindings with CRBN and target proteins and have the potential to recruit the target proteins to CRBN and induce their degradation.

**Fig 7:**
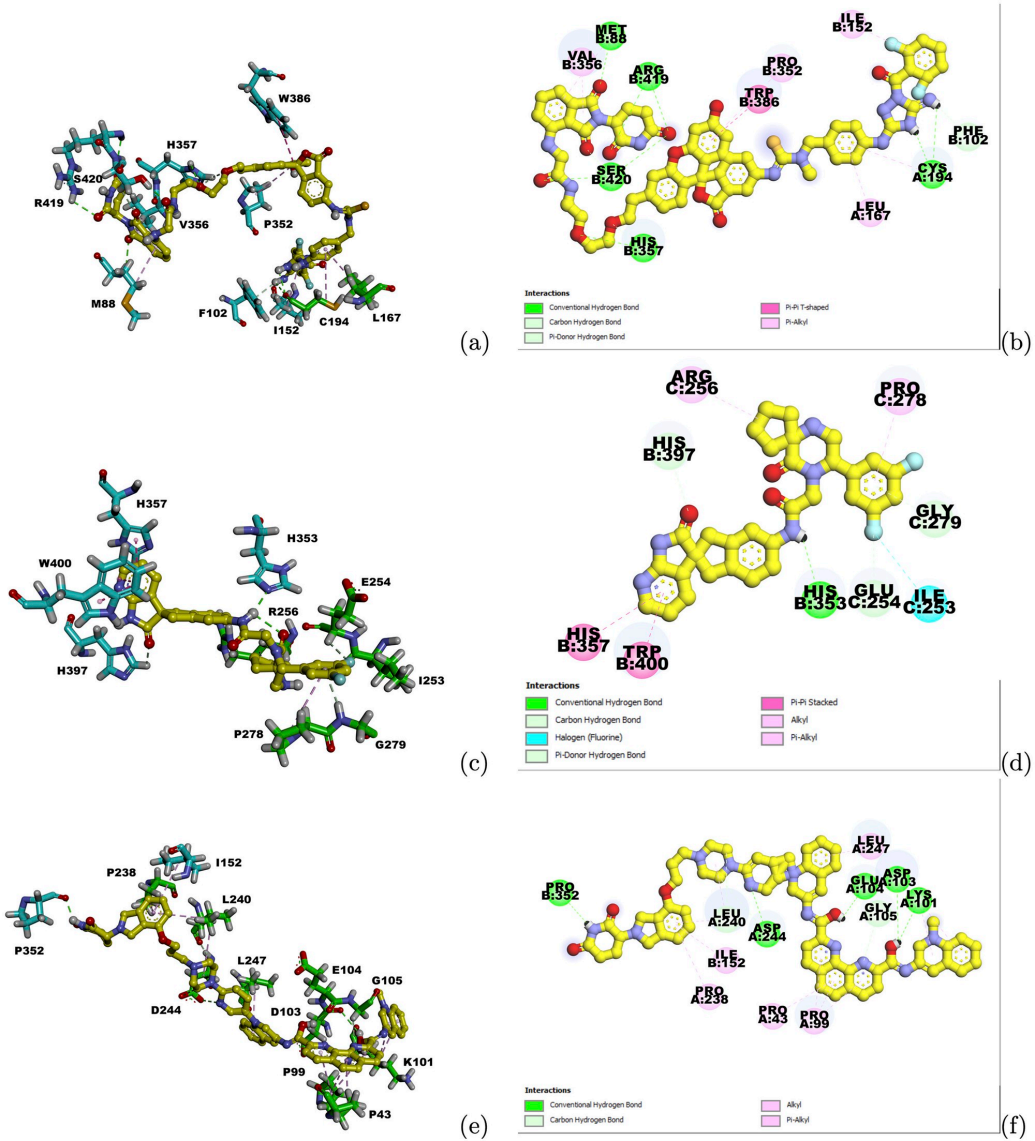
Interactions between predicted PROTACs and the CRBN-target protein complex structures. (a) Ligand-target interactions between the PROTAC and the complex structure for P12757. (b) 2D ligand-target interactions between the PROTAC and the complex structure for P12757. A and B represent the chain ID for P12757 and CRBN, respectively. (c) Ligand-target interactions between the PROTAC and the complex structure for Q9UER7. (d) 2D ligand-target interactions between the PROTAC and the complex structure for Q9UER7. B and C represent the chain ID for CRBN and Q9UER7, respectively. (e) Ligand-target interactions between the PROTAC and the complex structure for Q9H1I8. (f) 2D ligand-target interactions between the PROTAC and the complex structure for Q9H1I8. A and B represent the chain ID for Q9H1I8 and CRBN, respectively. Cyan sticks represent interacting residues on CRBN and green sticks represent interacting residues on the target protein. Yellow balls and sticks represent the ligand structure. Color representations for interaction types are shown in the figure legend.

After Autodock screening on the compounds from the Enamine and Medchem Express, ZINC000725236954, ZINC000103760984 and ZINC000029047404 were selected as the warheads for P12757, Q9UER7 and Q9H1I8, respectively, based on their docking scores. CRBN related moieties and linkers from PROTAC-DB were chosen to connect with the predicted warheads and formed new PROTACs which can dock into both binding pockets of CRBN and the target proteins in the complex structures. Interactions between these new PROTACs and the CRBN-target protein complex structures were shown in Fig 7. For P12757, PRO352 and TRP386 on CRBN still interacted with the CRBN moiety, while many other residues formed hydrogen bonding and hydrophobic interactions with the predicted PROTAC. ZINC000103760984 already occupied both binding pockets on CRBN and Q9UER7, functioning as a PROTAC on its own. HIS353 on CRBN formed hydrogen bonding interactions with this compound. For Q9H1I8, PRO352 maintained its interaction with CRBN moiety. These conserved interactions between the predicted PROTACs and CRBN-target complex structures suggested the potential of designing new PROTACs for these targets.

**Fig 8:**
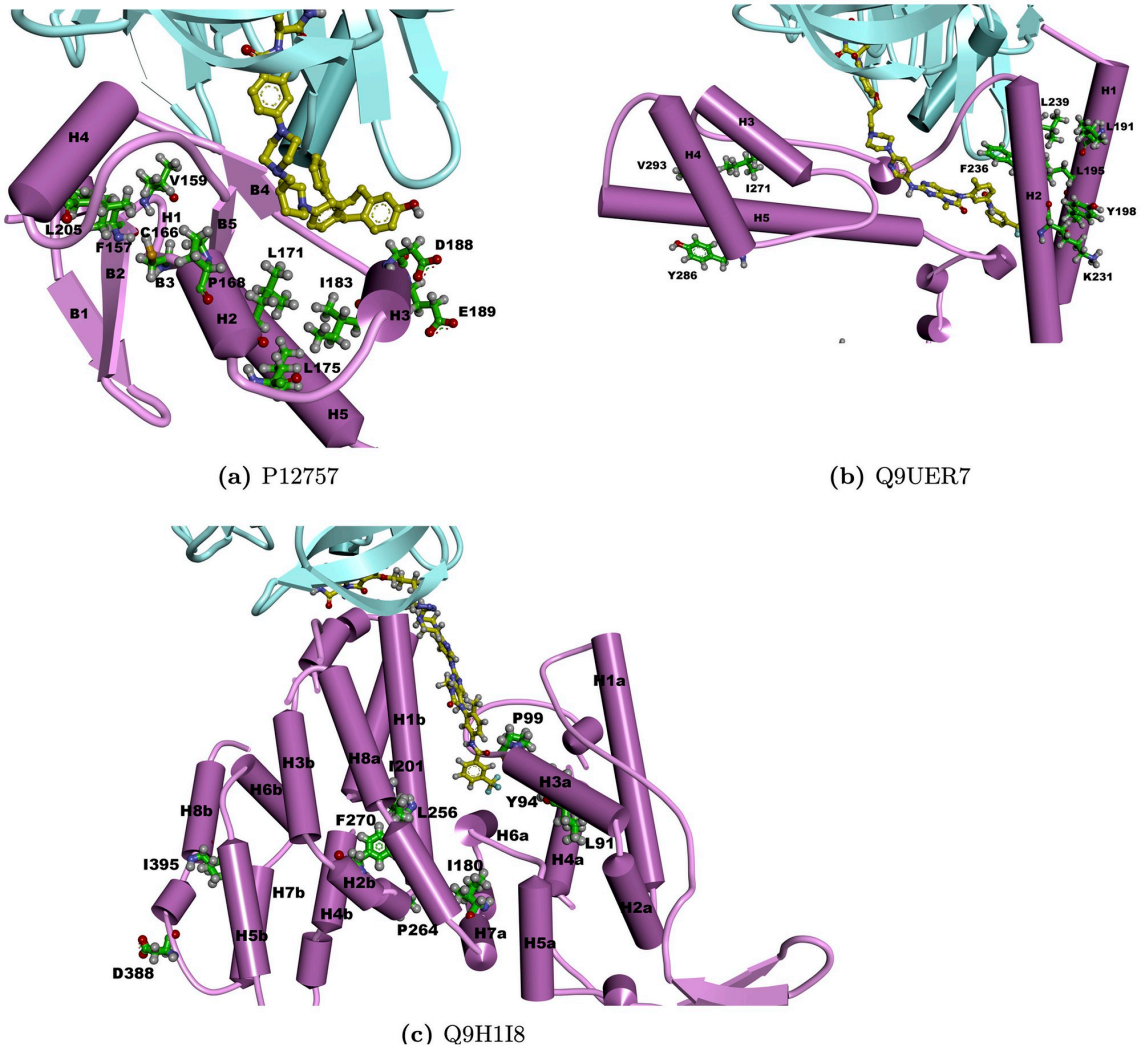
Structural mapping of the key positions for P12757, Q9UER7 and Q9H1I8. Purple colors represent CRBN and the target protein, respectively. Yellow and green balls and sticks represent predicted PROTACs and the top ranked positions, respectively. H and B represent *α* helix and *β* sheet, respectively.

Key positions were also identified for these three proteins through eSHAP analysis. In order to compare them with the docking results, the top ranked positions were mapped onto the modeled complex structures for P12757, Q9UER7 and Q9H1I8, as shown in Fig 8. The majority of these key positions were found around their PROTAC binding pockets on the modeled structures. Interestingly, the predicted PROTAC binding pockets for Q9H1I8 coincide with the interface between the human activating signal co-integrator complex (ASCC2) and ASCC3 [63]. LEU91, TYR94, PRO99, ILE 201, LEU256 and PHE270 are all around this PROTAC binding pockets and the ASCC3-ASCC2 interface. The identified key positions on these predicted model structures demonstrate that our model can provide valuable insights into the binding residues when designing PROTACs for understudied proteins.

## Conclusion

Protein degradation is an essential process involved in protein turnover within the cell. It provides a mechanism of quality control during protein folding, enables rapid response to cellular signals, and allows modulation of the pool of available amino acids. The majority of proteins undergo degradation through the ubiquitin–proteasome system, which involves a diverse group of proteins. The ubiquitin E3 ligase family is the largest family in ubiquitin signaling, comprising approximately 700 members with identified or predicted ligase activities, and plays a crucial role in ubiquitin signaling [64]. This family can be categorized into three subfamilies:: RING [65], HECT [66], and RING-Between-RING (RBR) E3 ligases [67], which include PARKIN and ARIH1. Mechanistically, RBR E3 ligases exhibit characteristics of both RING and HECT ligases. Currently, the majority of reported PROTACs rely on two E3 ligases, CRBN and VHL. Our training data also focus on the degradation data for the CRBN E3 ligase. It remains unknown if the model developed using CRBN can be applied to other E3 ligase. Studies on the protein kinase degradation showed that there are overlaps between CRBN-mediated degradation and VHL-mediated degradation, which indicates the targets degradable for one E3 ligase may also be potential targets for another E3 ligase.

Now, the majority of PROTACs are designed from known potent ligands that bind to well-studied proteins. However, many understudied proteins that could be potential drug targets have not been investigated using PROTACs. One of the most promising advantages of PROTACs lies in their ability to induce the degradation for these proteins. Our results regarding understudied human proteins can serve as a starting point for the development of PROTACs targeting these proteins. Based on our prediction results, further investigations such as sequence and structure comparisons, complex structure modeling, and linker modeling can be conducted and help to design PROTACs for these proteins.

Our method is the first machine learning method that utilizes protein sequence features to predict PROTAC-induced degradation for target proteins. In this study, we employed 23 different feature descriptors, three classification methods, and various combinations thereof to construct our machine learning models. These models were trained and validated by protein kinase degradation data and tested on other degradable, non-protein kinase proteins from PROTAC-DB. Extensive benchmark studies have shown that a RF model incorporating the ESM feature exhibits the best performance, while avoiding significant overfitting. The fully trained best model can be employed for PROTAC screening and predict possible target proteins which can be degraded by CRBN. Compared to other computational methods, his approach enables the screening of a large number of proteins in a short period. As more degradation data becomes available in the future, the accuracy of such models is expected to be greatly improved.

The limited availability of high-quality annotated data on PROTAC-induced degradation poses a significant challenge in the development of PROTAC machine learning models. In this study, experimentally determined protein kinase degradation data from Fischer’s group and additional data for proteins from other gene families in PROTAC-DB were used to train and evaluate our model. Despite the predominant presence of protein kinase degradation data in the available dataset, our model exhibits potential generalizability to other proteins due to its utilization of a pre-trained protein language model. This notion is further supported by external benchmark studies, where the testing proteins belonged to gene families distinct from those present in the training data. Moreover, as more PROTAC degradation data becomes accessible for various protein types in the future, the performance of our model can be enhanced even further.

In our current work, we focused on sequence information to construct and train our models. Incorporating structural features of the binding pockets for both E3 ligases and the target proteins could provide additional information and enhance the accuracy of PrePROTAC prediction. Current protein structure prediction methods, such as AlphaFold [68], are capable of generating model structures for proteins without known crystal structures. Nonetheless, careful selection of these model structures is essential, as they may contain randomly generated loops. It is anticipated that the inclusion of reliable structural features in our models will result in improved performance for PrePROTAC prediction.

## Methods

### Overview

The random forest (RF) and gradient boosting tree (GBT) classification methods, implemented in scikit-learn [69], were evaluated in this work and the one demonstrating the best performance was selected to predict whether a protein could be degraded by PROTAC-induced protein degradation. These models were trained using degradation data of human protein kinases from Fischer’s group [40] and tested on additional degradation data from PROTAC-DB [41]. Pre-trained features from iFeature [43], D-Script [70], and ESM [42] were selected as inputs for the RF and GBT classification methods. Different combinations of classification methods and features were tested using the cross-validation method on the training set, and the RF classification with the ESM feature exhibited the highest performance. The RF and GBT classification models, utilizing the ESM feature, were further evaluated on the external test set. Finally, the training set and test set were combined to construct the final PrePROTAC model.

### Training data set

One of the difficulties on PROTAC-induced protein degradation is few data available for systematic modeling studies. Comparing with traditional drug discovery, only a small number of PROTACs and targeted proteins are reported. Recently, Fischer’s group developed a large library of kinase-targeting degraders and identified degradable protein-kinases among human protein kinases and kinase-like proteins. [40]. Based on their results, 445 protein kinases were selected as the training set, including 201 proteins degraded by CRBN-recruiting degraders, 95 proteins degraded by VHL-recruiting degraders and 234 proteins which cannot be degraded by any degraders. There are some overlaps between proteins degraded by CRBN and VHL. For example, among the 95 proteins degraded by VHL, only 10 of them are exclusively degraded by VHL-recruiting degraders. Distributions of CRBN and VHL induced degradations in different protein kinase families was shown in Fig Z in S1 Text. Labels for the proteins in the training set are based on the experimental results. In the training set, if a target is degraded by CRBN, the target is labeled as positive, otherwise, negative. Since the majority of PROTAC targets are recruited by CRBN, machine learning models were trained for the CRBN targeted degradation.

### External test data set

In order to evaluate the machine learning classification model trained using the CRBN-protein kinase pairs, additional PROTAC-induced degradation data was collected from PROTAC-DB [41] to build an external test data set. PROTAC-DB is an online database which compiles information of PROTACs by searching PubMed publications with keywords of ‘degrader* or PROTAC or proteolysis targeting chimera’. 62 proteins were found to be degraded by CRBN or VHL-recruiting degraders and not included in protein kinase training set. Out of these, 8 proteins are VHL targets and it is unknown whether they can be degraded by CRBN. Four proteins belong to the receptor protein-tyrosine kinase family. The remaining 50 proteins were used as positive samples in the test data set. Sequence alignments using BlastP [49] were conducted on these proteins to explore their relationships with protein kinases in the training set.

There are few known negative PROTAC data. To incorporate negative controls into the test set, two groups of negative samples were generated based on the structural classifications from SCOP [47, 48]. The SCOP database provides various levels of protein structure classifications that reflect different structural and evolutionary relationships. The SCOP fold, which encompasses proteins in different superfamilies and families with the same topology, is the third level of the structural classification and used as the basis to select negative samples. One group consisted of 50 representative proteins randomly selected from the SCOP folds different with the degradable proteins in the test set and protein kinases in the training set. The other group comprised 50 randomly selected representative proteins in different SCOP super-families but within the same SCOP fold as the degradable proteins in the test set.

### Machine learning methods

The goal of this study is to identify the proteins that can be induced by PROTAC molecules and subsequently degraded by CRBN. Two different classification methods were investigated for this purpose, including RF and GBT classification methods. RF is a widely used classification algorithm that comprises multiple decision trees. Its underlying principle, known as “the wisdom of crowds,” makes it a simple yet powerful approach. On the other hand, GBT optimizes a cost function across the function space and combines weak learners into a single strong learner through iterative processes.

Here, these two different classification methods were trained to learn from the features describing proteins and distinguish between degradable and undegradable proteins in the training set. Limited by the size of the training data, four hyper-parameters were selected and tuned to reduce the over-fitting, including max_depth, min_samples_split, min_samples_leaf and n_estimators. The others were set to their default values in Scikit-learn. Max_depth defines the longest path between the root node and the leaf node. As the value increases, the accuracy may initially increase to a certain limit and then decrease due to overfitting. Setting this value appropriately is important to avoid overfitting. Min_samples_split decides the minimum number of samples for an internal node required to hold before being split into further nodes. required for an internal node before it can be split further. Increasing this value could limit the number of splits and help reduce overfitting, although setting it too large may lead to underfitting. Min_samples_leaf specifies the minimum number of samples that a node should have after getting split. It also helps to reduce overfitting. N_estimators determines the number of trees in the forest of the model. This parameter is closely related to the size of the data. These four Hyper-parameters were tuned using 5-fold grid-search cross validation method, and the ones with the best ROC-AUC scores were chosen for the next step of modeling.

### Pretrained features

Since there are only a few hundred data points available for PROTAC induced degradation, conducting large-scale training is not practical. Therefore, in this study, pre-trained features from other large-scale protein language modeling approaches were employed as initial inputs for the RF and GBT classification methods. These features include sequence features from iFeature [43], pre-trained protein-protein interaction (PPI) contact features from D-SCRIPT [44], and pre-trained protein sequence features from ESM [42]. These features are derived from a vast number of protein sequences or structures, and incorporating them as inputs for our classification models enables them to capture valuable information.

#### Sequence features from iFeature

We first tested the features based on structural and physio-chemical descriptors extracted from protein sequence data. iFeature [43] is a python-based toolkit which integrates 18 major sequence encoding schemes and 53 different types of feature descriptors. The number of features for these descriptors range from hundreds to thousands. In order to avoid over-fitting, 21 descriptors of them which contain less than 1000 features were selected as the features for the classification models. As shown in Table A in S1 Text, these descriptors can be separated into six groups, including amino acid composition related [71–74], grouped amino acid composition related, distribution of amino acid properties related [75] from AAindex database [76], amino acid distribution patterns related [77], conjoint triad descriptor related [78] and sequence-order feature related groups [79].

#### Contact features from D-SCRIPT

In PROTAC induced degradation, targeted proteins and E3 ligase will form complex structures. But such complex structures are different with the naturally formed protein-protein complex since PROTAC binds to the interface between them and hold them to stay together. That is why current PPI prediction methods cannot directly predict such pairs. However, the information hidden in the PPI is still useful for the prediction of PROTAC induced degradation because the formation of E3 ligase-target protein complex structures still follows basic physical-chemical principles. Thus, the pre-trained features used in the PPI prediction method can be a start point for PROTAC induced degradation.

In this work, the contact feature in D-SCRIPT [44], a deep learning method for predicting a physical interaction between two proteins given just their sequences, was applied. Starting from the pre-trained embedding models E1 and E2 which capture both local and global structural information from both sequences in one CRBN-target pair, a projection module was used to reduce the dimensions of E1 and E2 by using a fully-connected linear layer. A residue contact module was then used to model the interactions between the residues of each protein. This step generated a *n*-by-*m* contact prediction matrix which indicates the predicted probability that two residues (one in CRBN and the other one in target protein) are in contact. Here, *n* represents the sequence length of the CRBN and *m* represents the length of the target protein. This matrix was used as the initial features in our model. In order to get a fix-length array for each pair, a max pooling operation was used to change the matrix to *n*-dimensional array. This array represents *n*-dimensional contact features for each sample in the RF and GBT classification models.

#### Features from ESM-1b transformer

Recently, Facebook AI research group developed a deep contextual language model with unsupervised learning to train on 86 billion amino acids across 250 million protein sequences [42]. The resulting model contains information about biological properties in its representations. The representations are learned from sequence data alone, and ESM-1b model outperforms all tested single-sequence protein language models across a range of structure prediction tasks. Here, we used the output embedding features from ESM-1b model as the input features to train the RF and GBT classification models to identify PROTAC-induced degradable proteins.

### Performance evaluation

Performance of RF and GBT models with different features were evaluated by the repeated Stratified 5-Fold cross validation method. Area under the curve of Receiver operating characteristic curve (ROC-AUC) and precision score were calculated and compared between these models. The average precision (AP) score was calculated as the following AP score function (1):

$$\begin{array}{c} AP=\sum_{n}\left(R_{n}-R_{n-1}\right)P_{n}\; \end{array}$$ (1)

where Rn and Pn are the precision and recall at the nth threshold. The AP score estimates the area under the Precision-Recall curve. The model with the highest ROC-AUC and AP score was selected for further prediction.

### eSHAP analysis of PrePROTAC model identifies key residues contributing to PROTAC activities

SHAP (SHapley Additive exPlanations) [80] [81] [82] values are widely used to explain machine learning models. TreeExplainer in the shap python package was applied to get the SHAP values for the features used in the PrePROTAC model. The 20 embedding features with the highest SHAP values were selected and these features should play important roles in the prediction of our model. Since these features are embedding attributes, their specific meaning is unknown. In order to investigate which residues on the protein make key contributions to PROTAC activity, an in silico mutagenesis was performed on the proteins in the training set. Each residue was mutated sequentially once a time to the amino acid with the opposite property. For example positive charged amino acids mutated to negative charged amino acids and polar amino acids mutated to hydrophobic amino acids. For each position on the protein sequence, the differences of the 20 selected embedding features between the mutated sequences and the original sequence was calculated according to the following formula:

$$\begin{array}{c} Difference\_score\left(x\right)=sqrt(\sum_{n=1}^{20}{\left(f_{n}^{ref}\left(x\right)-f_{n}^{mutated}\left(x\right)\right)}^{2})\; \end{array}$$ (2)

The importance of all the amino acid positions were measured by the difference scores. This embedding SHAP (eSHAP) analysis could help identify the residues which contribute most on the selected features. The bigger the difference score is, the higher impact of this position has on these features. The positions with the top-ranked difference scores were selected as the key positions for this protein.

### PrePROTAC prediction on understudied disease associated human proteins

It is well known that only a subset of the human genome that is considered druggable in terms of drug-like small molecules. There is only limited information for the understudied disease proteins [83]. Hence, we built an understudied human protein database by removing the druggable proteins (Tclinic and Tchem) in Pharos [84] and Casas’s druggable proteins [85] from DisGeNet, a human disease associated genome database [86]. DisGeNet includes 12,475 human proteins which are related with certain diseases but have few ligand binding information. The PrePROTAC model was applied to these proteins to obtain the probability score of being degraded by CRBN.

### Protein-protein docking and protein-ligand docking

In order to design potential PROTACs for the target proteins, protein-protein docking and protein-ligand docking methods were applied on target proteins to build complex structures between E3-ligases and target proteins, and to predict PROTACs which were able to interact with these target proteins. Zdock [87] was firstly performed to bring CRBN and target proteins together. The interface residues of CRBN were used to guide the protein-protein docking in Zdock. A local protein-protein docking protocol in Rosetta [88] was used to refine the complex structure obtained from Zdock. Then, the CRBN-target protein complex structure was decided by choosing the complex structure with the lowest energy. Autodock [89] was then applied to 2,074 CRBN related PROTACs in PROTAC-DB to select the PROTAC with the best docking score and docking conformation for each target. Furthermore, in order to identify additional PROTACs for these target proteins beyond the existing PROTACs in PROTAC-DB, two other compound databases were considered for warhead screening, including 1,920 fragment-based compounds from high fidelity fragment library of Enamine [60] and 2,016 drugs provided by MedChem Express, LLC (Monmouth Junction, NJ, USA) in ZINC database [61]. Compounds were docked on the target protein via Autodock. The compounds with the best docking scores were used as the warheads for the targets protein and connected with the CRBN related E3 ligase moieties through the linkers provided in PROTAC-DB. The binding pocket of the co-crystallized ligand in CRBN (Lenalidomide in 4TZ4) and the predicted binding pockets on the target proteins were used as the reference binding pockets. These compounds which can bind to both binding pockets were selected as the predicted PROTACs for the target proteins.

## Supporting information

S1 Text Supporting information for methods and results. Supplemental materials for PrePROTAC model training, testing and prediction. Additional results for eSHAP analysis on the human protein kinases.
(PDF)

## References

[1] Hopkins AL, Groom CR. The druggable genome. Nature Reviews Drug Discovery. 2002;1(9):727–730. doi: 10.1038/nrd892 12209152

[2] Overington JP, Al-Lazikani B, Hopkins AL. How many drug targets are there? Nature Reviews Drug Discovery. 2006;5(12):993–996. doi: 10.1038/nrd2199 17139284

[3] Lazo JS, Sharlow ER. Drugging Undruggable Molecular Cancer Targets. Annual Review of Pharmacology and Toxicology. 2016;56:23–40. doi: 10.1146/annurev-pharmtox-010715-103440 26527069

[4] Nalawansha DA, Crews CM. PROTACs: An Emerging Therapeutic Modality in Precision Medicine. Cell Chemical Biology. 2020;27(8):998–1014. doi: 10.1016/j.chembiol.2020.07.020 32795419 PMC9424844

[5] Paiva SL, Crews CM. Targeted protein degradation: elements of PROTAC design. Current Opinion in Chemical Biology. 2019;50:111–119. doi: 10.1016/j.cbpa.2019.02.022 31004963 PMC6930012

[6] Smith BE, Wang SL, Jaime-Figueroa S, Harbin A, Wang J, Hamman BD, et al. Differential PROTAC substrate specificity dictated by orientation of recruited E3 ligase. Nature Communications. 2019;10(1). doi: 10.1038/s41467-018-08027-7 30631068 PMC6328587

[7] Gechijian LN, Buckley DL, Lawlor MA, Reyes JM, Paulk J, Ott CJ, et al. Functional TRIM24 degrader via conjugation of ineffectual bromodomain and VHL ligands. Nature Chemical Biology. 2018;14(4):405–412. doi: 10.1038/s41589-018-0010-y 29507391 PMC5866761

[8] Bassi ZI, Fillmore MC, Miah AH, Chapman TD, Maller C, Roberts EJ, et al. Modulating PCAF/GCN5 Immune Cell Function through a PROTAC Approach. ACS Chemical Biology. 2018;13(10):2862–2867. doi: 10.1021/acschembio.8b00705 30200762

[9] Cromm PM, Samarasinghe KTG, Hines J, Crews CM. Addressing Kinase-Independent Functions of Fak via PROTAC-Mediated Degradation. Journal of the American Chemical Society. 2018;140(49):17019–17026. doi: 10.1021/jacs.8b08008 30444612

[10] Degorce SL, Tavana O, Banks E, Crafter C, Gingipalli L, Kouvchinov D, et al. Discovery of Proteolysis-Targeting Chimera Molecules that Selectively Degrade the IRAK3 Pseudokinase. Journal of Medicinal Chemistry. 2020;63(18):10460–10473. doi: 10.1021/acs.jmedchem.0c01125 32803978

[11] Crews CM. Targeting the Undruggable Proteome: The Small Molecules of My Dreams. Chemistry & Biology. 2010;17(6):551–555. doi: 10.1016/j.chembiol.2010.05.011 20609404 PMC2925121

[12] Schapira M, Calabrese MF, Bullock AN, Crews CM. Targeted protein degradation: expanding the toolbox. Nature Reviews Drug Discovery. 2019;18(12):949–963. doi: 10.1038/s41573-019-0047-y 31666732

[13] Lai AC, Crews CM. Induced protein degradation: an emerging drug discovery paradigm. Nature Reviews Drug Discovery. 2016;16(2):101–114. doi: 10.1038/nrd.2016.211 27885283 PMC5684876

[14] Bondeson DP, Smith BE, Burslem GM, Buhimschi AD, Hines J, Jaime-Figueroa S, et al. Lessons in PROTAC Design from Selective Degradation with a Promiscuous Warhead. Cell Chemical Biology. 2018;25(1):78–87.e5. doi: 10.1016/j.chembiol.2017.09.010 29129718 PMC5777153

[15] Gechijian LN, Buckley DL, Lawlor MA, Reyes JM, Paulk J, Ott CJ, et al. Functional TRIM24 degrader via conjugation of ineffectual bromodomain and VHL ligands. Nature Chemical Biology. 2018;14(4):405–412. doi: 10.1038/s41589-018-0010-y 29507391 PMC5866761

[16] Burslem GM, Smith BE, Lai AC, Jaime-Figueroa S, McQuaid DC, Bondeson DP, et al. The Advantages of Targeted Protein Degradation Over Inhibition: An RTK Case Study. Cell Chemical Biology. 2018;25(1):67–77.e3. doi: 10.1016/j.chembiol.2017.09.009 29129716 PMC5831399

[17] Cromm PM, Samarasinghe KTG, Hines J, Crews CM. Addressing Kinase-Independent Functions of Fak via PROTAC-Mediated Degradation. Journal of the American Chemical Society. 2018;140(49):17019–17026. doi: 10.1021/jacs.8b08008 30444612

[18] Popow J, Arnhof H, Bader G, Berger H, Ciulli A, Covini D, et al. Highly Selective PTK2 Proteolysis Targeting Chimeras to Probe Focal Adhesion Kinase Scaffolding Functions. Journal of Medicinal Chemistry. 2019;62(5):2508–2520. doi: 10.1021/acs.jmedchem.8b01826 30739444

[19] Vogelmann A, Robaa D, Sippl W, Jung M. Proteolysis targeting chimeras (PROTACs) for epigenetics research. Current Opinion in Chemical Biology. 2020;57:8–16. doi: 10.1016/j.cbpa.2020.01.010 32146413

[20] Winter GE, Buckley DL, Paulk J, Roberts JM, Souza A, Dhe-Paganon S, et al. Phthalimide conjugation as a strategy for in vivo target protein degradation. Science. 2015;348(6241):1376–1381. doi: 10.1126/science.aab1433 25999370 PMC4937790

[21] Gadd MS, Testa A, Lucas X, Chan KH, Chen W, Lamont DJ, et al. Structural basis of PROTAC cooperative recognition for selective protein degradation. Nature Chemical Biology. 2017;13(5):514–521. doi: 10.1038/nchembio.2329 28288108 PMC5392356

[22] Raina K, Lu J, Qian Y, Altieri M, Gordon D, Rossi AMK, et al. PROTAC-induced BET protein degradation as a therapy for castration-resistant prostate cancer. Proceedings of the National Academy of Sciences. 2016;113(26):7124–7129. doi: 10.1073/pnas.1521738113 27274052 PMC4932933

[23] Schiedel M, Herp D, Hammelmann S, Swyter S, Lehotzky A, Robaa D, et al. Chemically Induced Degradation of Sirtuin 2 (Sirt2) by a Proteolysis Targeting Chimera (PROTAC) Based on Sirtuin Rearranging Ligands (SirReals). Journal of Medicinal Chemistry. 2017;61(2):482–491. doi: 10.1021/acs.jmedchem.6b01872 28379698

[24] An Z, Lv W, Su S, Wu W, Rao Y. Developing potent PROTACs tools for selective degradation of HDAC6 protein. Protein & Cell. 2019;10(8):606–609. doi: 10.1007/s13238-018-0602-z 30603959 PMC6626596

[25] Smalley JP, Adams GE, Millard CJ, Song Y, Norris JKS, Schwabe JWR, et al. PROTAC-mediated degradation of class I histone deacetylase enzymes in corepressor complexes. Chemical Communications. 2020;56(32):4476–4479. doi: 10.1039/d0cc01485k 32201871 PMC7610821

[26] Dong H, Liu S, Zhang X, Chen S, Kang L, Chen Y, et al. An Allosteric PRC2 Inhibitor Targeting EED Suppresses Tumor Progression by Modulating the Immune Response. Cancer Research. 2019;79(21):5587–5596. doi: 10.1158/0008-5472.CAN-19-0428 31395608

[27] Hsu JHR, Rasmusson T, Robinson J, Pachl F, Read J, Kawatkar S, et al. EED-Targeted PROTACs Degrade EED, EZH2, and SUZ12 in the PRC2 Complex. Cell Chemical Biology. 2020;27(1):41–46.e17. doi: 10.1016/j.chembiol.2019.11.004 31786184

[28] Potjewyd F, Turner AMW, Beri J, Rectenwald JM, Norris-Drouin JL, Cholensky SH, et al. Degradation of Polycomb Repressive Complex 2 with an EED-Targeted Bivalent Chemical Degrader. Cell Chemical Biology. 2020;27(1):47–56.e15. doi: 10.1016/j.chembiol.2019.11.006 31831267 PMC7004250

[29] Salami J, Alabi S, Willard RR, Vitale NJ, Wang J, Dong H, et al. Androgen receptor degradation by the proteolysis-targeting chimera ARCC-4 outperforms enzalutamide in cellular models of prostate cancer drug resistance. Communications Biology. 2018;1(1). doi: 10.1038/s42003-018-0105-8 30271980 PMC6123676

[30] Buhimschi AD, Armstrong HA, Toure M, Jaime-Figueroa S, Chen TL, Lehman AM, et al. Targeting the C481S Ibrutinib-Resistance Mutation in Bruton’s Tyrosine Kinase Using PROTAC-Mediated Degradation. Biochemistry. 2018;57(26):3564–3575. doi: 10.1021/acs.biochem.8b00391 29851337

[31] Mares A, Miah AH, Smith IED, Rackham M, Thawani AR, Cryan J, et al. Extended pharmacodynamic responses observed upon PROTAC-mediated degradation of RIPK2. Communications Biology. 2020;3(1). doi: 10.1038/s42003-020-0868-6 32198438 PMC7083851

[32] Zaidman D, Prilusky J, London N. PRosettaC: Rosetta Based Modeling of PROTAC Mediated Ternary Complexes. Journal of Chemical Information and Modeling. 2020;60(10):4894–4903. doi: 10.1021/acs.jcim.0c00589 32976709 PMC7592117

[33] Bai N, Miller SA, Andrianov GV, Yates M, Kirubakaran P, Karanicolas J. Rationalizing PROTAC-Mediated Ternary Complex Formation Using Rosetta. Journal of Chemical Information and Modeling. 2021;61(3):1368–1382. doi: 10.1021/acs.jcim.0c01451 33625214 PMC8866032

[34] Nowak RP, DeAngelo SL, Buckley D, He Z, Donovan KA, An J, et al. Plasticity in binding confers selectivity in ligand-induced protein degradation. Nature Chemical Biology. 2018;14(7):706–714. doi: 10.1038/s41589-018-0055-y 29892083 PMC6202246

[35] Drummond ML, Williams CI. In Silico Modeling of PROTAC-Mediated Ternary Complexes: Validation and Application. Journal of Chemical Information and Modeling. 2019;59(4):1634–1644. doi: 10.1021/acs.jcim.8b00872 30714732

[36] Drummond ML, Henry A, Li H, Williams CI. Improved Accuracy for Modeling PROTAC-Mediated Ternary Complex Formation and Targeted Protein Degradation via New In Silico Methodologies. Journal of Chemical Information and Modeling. 2020;60(10):5234–5254. doi: 10.1021/acs.jcim.0c00897 32969649

[37] Lebraud H, Wright DJ, Johnson CN, Heightman TD. Protein Degradation by In-Cell Self-Assembly of Proteolysis Targeting Chimeras. ACS Central Science. 2016;2(12):927–934. doi: 10.1021/acscentsci.6b00280 28058282 PMC5200928

[38] Testa A, Hughes SJ, Lucas X, Wright JE, Ciulli A. Structure-Based Design of a Macrocyclic PROTAC. Angewandte Chemie International Edition. 2019;59(4):1727–1734. doi: 10.1002/anie.201914396 31746102 PMC7004083

[39] Imrie F, Bradley AR, van der Schaar M, Deane CM. Deep Generative Models for 3D Linker Design. Journal of Chemical Information and Modeling. 2020;60(4):1983–1995. doi: 10.1021/acs.jcim.9b01120 32195587 PMC7189367

[40] Donovan KA, Ferguson FM, Bushman JW, Eleuteri NA, Bhunia D, Ryu S, et al. Mapping the Degradable Kinome Provides a Resource for Expedited Degrader Development. cell. 2020;183(6):1714–1731. doi: 10.1016/j.cell.2020.10.038 33275901 PMC10294644

[41] Weng G, Shen C, Cao D, Gao J, Dong X, He Q, et al. PROTAC-DB: an online database of PROTACs. Nucleic Acids Research. 2021;49(D1):D1381–D1387. doi: 10.1093/nar/gkaa807 33010159 PMC7778940

[42] Rives A, Meier J, Sercu T, Goyal S, Lin Z, Liu J, et al. Biological structure and function emerge from scaling unsupervised learning to 250 million protein sequences. Proceedings of the National Academy of Sciences of the United States of America. 2021;118(15):e2016239118. doi: 10.1073/pnas.2016239118 33876751 PMC8053943

[43] Chen Z, Zhao P, Li F, Leier A, Marquez-Lago TT, Wang Y, et al. iFeature: a Python package and web server for features extraction and selection from protein and peptide sequences. Bioinformatics. 2018;34(14):2499–2502. doi: 10.1093/bioinformatics/bty140 29528364 PMC6658705

[44] Sledzieski S, Singh R, Cowen L, Berger B. Sequence-based prediction of protein-protein interactions: a structure-aware interpretable deep learning model. biorxiv. 2021;. 10.1016/j.cels.2021.08.010 PMC8586911 34536380

[45] Zhang QC, Petrey D, Garzón JI, Deng L, Honig B. PrePPI: a structure-informed database of protein–protein interactions. Nucleic acids research. 2012;41(D1):D828–D833. doi: 10.1093/nar/gks1231 23193263 PMC3531098

[46] Petrey D, Zhao H, Trudeau SJ, Murray D, Honig B. PrePPI: A Structure Informed Proteome-wide Database of Protein–Protein Interactions. Journal of Molecular Biology. 2023; p. 168052. doi: 10.1016/j.jmb.2023.168052 36933822 PMC10293085

[47] Andreeva A, Howorth D, Chothia C, Kulesha E, Murzin AG. SCOP2 prototype: a new approach to protein structure mining. Nucleic Acids Research. 2013;42(D1):D310–D314. doi: 10.1093/nar/gkt1242 24293656 PMC3964979

[48] Andreeva A, Kulesha E, Gough J, Murzin AG. The SCOP database in 2020: expanded classification of representative family and superfamily domains of known protein structures. Nucleic Acids Research. 2019;48(D1):D376–D382. doi: 10.1093/nar/gkz1064 PMC7139981 31724711

[49] Camacho C, Coulouris G, Avagyan V, Ma N, Papadopoulos J, Bealer K, et al. BLAST+: architecture and applications. BMC bioinformatics. 2009;10:1–9. doi: 10.1186/1471-2105-10-421 20003500 PMC2803857

[50] Modi V, Dunbrack RL. A structurally-validated multiple sequence alignment of 497 human protein kinase domains. Scientific reports. 2019;9(1):1–16. doi: 10.1038/s41598-019-56499-4 31875044 PMC6930252

[51] Kumar S, Stecher G, Li M, Knyaz C, Tamura K. MEGA X: molecular evolutionary genetics analysis across computing platforms. Molecular biology and evolution. 2018;35(6):1547. doi: 10.1093/molbev/msy096 29722887 PMC5967553

[52] Letunic I, Bork P. Interactive tree of life (iTOL) v3: an online tool for the display and annotation of phylogenetic and other trees. Nucleic acids research. 2016;44(W1):W242–W245. doi: 10.1093/nar/gkw290 27095192 PMC4987883

[53] Hanks SK, Quinn AM, Hunter T. The protein kinase family: conserved features and deduced phylogeny of the catalytic domains. Science. 1988;241(4861):42–52. doi: 10.1126/science.3291115 3291115

[54] Manning G, Whyte DB, Martinez R, Hunter T, Sudarsanam S. The protein kinase complement of the human genome. Science. 2002;298(5600):1912–1934. doi: 10.1126/science.1075762 12471243

[55] Lu J, Qian Y, Altieri M, Dong H, Wang J, Raina K, et al. Hijacking the E3 ubiquitin ligase cereblon to efficiently target BRD4. Chemistry & biology. 2015;22(6):755–763. doi: 10.1016/j.chembiol.2015.05.009 26051217 PMC4475452

[56] Qu J, Nakamura T, Cao G, Holland EA, McKercher SR, Lipton SA. S-Nitrosylation activates Cdk5 and contributes to synaptic spine loss induced by *β*-amyloid peptide. Proceedings of the National Academy of Sciences. 2011;108(34):14330–14335. doi: 10.1073/pnas.1105172108 21844361 PMC3161554

[57] Haun F, Nakamura T, Shiu AD, Cho DH, Tsunemi T, Holland EA, et al. S-nitrosylation of dynamin-related protein 1 mediates mutant huntingtin-induced mitochondrial fragmentation and neuronal injury in Huntington’s disease. Antioxidants & redox signaling. 2013;19(11):1173–1184. doi: 10.1089/ars.2012.4928 23641925 PMC3785802

[58] Walter S, Atzmon G, Demerath EW, Garcia ME, Kaplan RC, Kumari M, et al. A genome-wide association study of aging. Neurobiology of aging. 2011;32(11):2109–e15. doi: 10.1016/j.neurobiolaging.2011.05.026 21782286 PMC3193030

[59] Castillo E, Leon J, Mazzei G, Abolhassani N, Haruyama N, Saito T, et al. Comparative profiling of cortical gene expression in Alzheimer’s disease patients and mouse models demonstrates a link between amyloidosis and neuroinflammation. Scientific reports. 2017;7(1):1–16. doi: 10.1038/s41598-017-17999-3 29259249 PMC5736730

[60] Grygorenko OO. Enamine Ltd.: The Science and Business of Organic Chemistry and Beyond; 2021.

[61] Sterling T, Irwin JJ. ZINC 15–ligand discovery for everyone. Journal of chemical information and modeling. 2015;55(11):2324–2337. doi: 10.1021/acs.jcim.5b00559 26479676 PMC4658288

[62] SYSTÈMES D. BIOVIA Discovery Studio; 2016. Available from: http://accelrys.com/products/collaborative-science/biovia-discovery-studio/.

[63] Jia J, Absmeier E, Holton N, Pietrzyk-Brzezinska AJ, Hackert P, Bohnsack KE, et al. The interaction of DNA repair factors ASCC2 and ASCC3 is affected by somatic cancer mutations. Nature communications. 2020;11(1):1–13. doi: 10.1038/s41467-020-19221-x 33139697 PMC7608686

[64] Li W, Bengtson MH, Ulbrich A, Matsuda A, Reddy VA, Orth A, et al. Genome-Wide and Functional Annotation of Human E3 Ubiquitin Ligases Identifies MULAN, a Mitochondrial E3 that Regulates the Organelle’s Dynamics and Signaling. PLoS ONE. 2008;3(1):e1487. doi: 10.1371/journal.pone.0001487 18213395 PMC2198940

[65] Deshaies RJ, Joazeiro CAP. RING Domain E3 Ubiquitin Ligases. Annual Review of Biochemistry. 2009;78(1):399–434. doi: 10.1146/annurev.biochem.78.101807.093809 19489725

[66] Berndsen CE, Wolberger C. New insights into ubiquitin E3 ligase mechanism. Nature Structural & Molecular Biology. 2014;21(4):301–307. doi: 10.1038/nsmb.2780 24699078

[67] Spratt DE, Walden H, Shaw GS. RBR E3 ubiquitin ligases: new structures, new insights, new questions. Biochemical Journal. 2014;458(3):421–437. doi: 10.1042/BJ20140006 24576094 PMC3940038

[68] Jumper J, Evans R, Pritzel A, Green T, Figurnov M, Ronneberger O, et al. Highly accurate protein structure prediction with AlphaFold. Nature. 2021;596(7873):583–589. doi: 10.1038/s41586-021-03819-2 34265844 PMC8371605

[69] Pedregosa F, Varoquaux G, Gramfort A, Michel V, Thirion B, Grisel O, et al. Scikit-learn: Machine Learning in Python. Journal of Machine Learning Research. 2011;12:2825–2830.

[70] Sledzieski S, Singh R, Cowen L, Berger B. D-SCRIPT translates genome to phenome with sequence-based, structure-aware, genome-scale predictions of protein-protein interactions. Cell Systems. 2021;12(10):969–982. doi: 10.1016/j.cels.2021.08.010 34536380 PMC8586911

[71] Chen X, Qiu JD, Shi SP, Suo SB, Huang SY, Liang RP. Incorporating key position and amino acid residue features to identify general and species-specific Ubiquitin conjugation sites. Bioinformatics. 2013;29(13):1614–1622. doi: 10.1093/bioinformatics/btt196 23626001

[72] Liu LM, Xu Y, Chou KC. iPGK-PseAAC: Identify Lysine Phosphoglycerylation Sites in Proteins by Incorporating Four Different Tiers of Amino Acid Pairwise Coupling Information into the General PseAAC. Medicinal Chemistry. 2017;13(6):552–559. doi: 10.2174/1573406413666170515120507 28521678

[73] Saravanan V, Gautham N. Harnessing Computational Biology for Exact Linear B-Cell Epitope Prediction: A Novel Amino Acid Composition-Based Feature Descriptor. OMICS. 2015;19(10):648–658. doi: 10.1089/omi.2015.0095 26406767

[74] Bhasin M, Raghava GPS. Classification of nuclear receptors based on amino acid composition and dipeptide composition. Journal of Biological Chemistry. 2004;279(22):23262–23266. doi: 10.1074/jbc.M401932200 15039428

[75] Sokal RR, Thomson BA. Population structure inferred by local spatial autocorrelation: an example from an Amerindian tribal population. The American Journal of Physical Anthropology. 2006;129(1):121–131. doi: 10.1002/ajpa.20250 16261547

[76] Kawashima S, Pokarowski P, Pokarowska M, Kolinski A, Katayama T, Kanehisa M. AAindex: amino acid index database, progress report 2008. Nucleic Acids Research. 2008;36:D202–D205. doi: 10.1093/nar/gkm998 17998252 PMC2238890

[77] Dubchak I, Muchnikt I, Holbrook SR, hou Kim S. Prediction of protein folding class using global description of amino acid sequence. Proceedings of the National Academy of Sciences of the United States of America. 1995;92(19):8700–8704. doi: 10.1073/pnas.92.19.8700 7568000 PMC41034

[78] Shen J, Zhang J, Luo X, Zhu W, Yu K, Chen K, et al. Predicting protein-protein interactions based only on sequences information. Proceedings of the National Academy of Sciences of the United States of America. 2007;104(11):4337–4341. doi: 10.1073/pnas.0607879104 17360525 PMC1838603

[79] Chou KC, Cai YD. Prediction of protein subcellular locations by GO-FunD-PseAA predictor. Biochemical and Biophysical Research Communications. 2004;320(4):1236–1239. doi: 10.1016/j.bbrc.2004.06.073 15249222

[80] Štrumbelj E, Kononenko I. Explaining prediction models and individual predictions with feature contributions. Knowledge and information systems. 2014;41(3):647–665. doi: 10.1007/s10115-013-0679-x

[81] Lundberg SM, Lee SI. A unified approach to interpreting model predictions. Advances in neural information processing systems. 2017;30.

[82] Lundberg SM, Erion G, Chen H, DeGrave A, Prutkin JM, Nair B, et al. From local explanations to global understanding with explainable AI for trees. Nature machine intelligence. 2020;2(1):56–67. doi: 10.1038/s42256-019-0138-9 32607472 PMC7326367

[83] Finan C, Gaulton A, Kruger FA, Lumbers RT, Shah T, Engmann J, et al. The druggable genome and support for target identification and validation in drug development. Science translational medicine. 2017;9(383). doi: 10.1126/scitranslmed.aag1166 28356508 PMC6321762

[84] Sheils TK, Mathias SL, Kelleher KJ, Siramshetty VB, Nguyen DT, Bologa CG, et al. TCRD and Pharos 2021: mining the human proteome for disease biology. Nucleic Acids Research. 2020;49(D1):D1334–D1346. doi: 10.1093/nar/gkaa993 PMC7778974 33156327

[85] Finan C, Gaulton A, Kruger FA, Lumbers RT, Shah T, Engmann J, et al. The druggable genome and support for target identification and validation in drug development. Science Translational Medicine. 2017;9:eaag1166. doi: 10.1126/scitranslmed.aag1166 28356508 PMC6321762

[86] Piñero J, Ramírez-Anguita JM, Saüch-Pitarch J, Ronzano F, Centeno E, Sanz F, et al. The DisGeNET knowledge platform for disease genomics: 2019 update. Nucleic Acids Research. 2020;48(D1):D845–D855. doi: 10.1093/nar/gkz1021 31680165 PMC7145631

[87] Pierce BG, Wiehe K, Hwang H, Kim BH, Vreven T, Weng Z. ZDOCK server: interactive docking prediction of protein–protein complexes and symmetric multimers. Bioinformatics. 2014;30(12):1771–1773. doi: 10.1093/bioinformatics/btu097 24532726 PMC4058926

[88] Sircar A, Chaudhury S, Kilambi KP, Berrondo M, Gray JJ. A generalized approach to sampling backbone conformations with RosettaDock for CAPRI rounds 13–19. Proteins: Structure, Function, and Bioinformatics. 2010;78(15):3115–3123. doi: 10.1002/prot.22765 20535822 PMC2952725

[89] Forli S, Huey R, Pique ME, Sanner MF, Goodsell DS, Olson AJ. Computational protein–ligand docking and virtual drug screening with the AutoDock suite. Nature protocols. 2016;11(5):905–919. doi: 10.1038/nprot.2016.051 27077332 PMC4868550

