# Supplementary material for: Elucidation of genome-wide understudied proteins targeted by PROTAC-induced degradation using interpretable machine learning
Source: PLoS Comput Biol. 2023 Aug 17;19(8):e1010974. doi: 10.1371/journal.pcbi.1010974 (PMC10464998; doi:10.1371/journal.pcbi.1010974)
Supplement: S1 Text — Supplemental materials for PrePROTAC model training, testing and prediction. Additional results for eSHAP analysis on the human protein kinases. (PDF) [file pcbi.1010974.s001.pdf]

# - **Supplemental Materials** -

Elucidation of Genome-wide Understudied Proteins targeted by PROTAC-induced degradation using Interpretable Machine Learning

Li Xie[1] and Lei Xie[1,2,3,*]

[1]*Department of Computer Science, Hunter College, The City University of New York, New York, 10065, United States of America*

[2]*Ph.D. Program in Computer Science, The Graduate Center, The City University of New York, New York, 10016, United States of America*

[3]*Helen and Robert Appel Alzheimer's Disease Research Institute, Feil Family Brain & Mind Research Institute, Weill Cornell Medicine, Cornell University, New York, 10021, United States of America*

[*]*lei.xie@hunter.cuny.edu*

1 Methods and Results

1.1 Hyper-parameter tuning of random forest and gradient boosting classification methods for different features

For each set of features, 5-fold grid-search cross validation method was used to get the optimized hyper-parameters for random forest and gradient boosting classification methods. Limited by the size of the training data, four hyper-parameters were selected and tuned, including max_depth, min_samples_split, min_samples_leaf and n_estimators, the others were set as the default values in Scikit-learn. Max_depth defines the longest path between the root node and the leaf node. When the value increases, the accuracy could increase to a certain limit and then decrease due to the over-fitting of the data. It is important to set this value appropriately to avoid over-fitting. Min_samples_split decides the minimum number of samples for an internal node required to hold before being split into further nodes. Increasing this value could limit the number of splits and help reduce over-fitting. But too large value could cause under-fitting. Min_samples_leaf specifies the minimum number of samples that a node should have after getting split. It also helps to reduce overfitting. N_estimators decides the number of trees in the forest of the model. This parameter is largely correlated to the size of the data. The values of these hyper-parameters used in the random forest classification model with different features were listed in Table A and hyper-parameters for the gradient boosting model were listed in Table B.

Table A. Optimized hyper-parameters for the random forest classification models with different features

Hyper-parameters for the random forest classification models				
Feature	max_depth	min_samples_split	min_samples_leaf	n_estimators
Amino acid composition related				
AAC	10	2	2	300
CKSAAP	7	4	1	400
TPC	9	8	4	200
DPC	7	4	2	300
DDE	10	10	1	200
PAAC	10	4	1	400
APAAC	8	2	1	200
Grouped amino acid composition related				
GAAC	10	4	2	100
CKSAAGP	8	6	2	200
GDPC	10	4	2	100
GTPC	8	2	4	200
Distribution of amino acid properties related				
Moran	9	4	2	200
Geary	9	6	1	400
NMBroto	8	2	3	100
Amino acid distribution patterns related				
CTDC	9	2	1	300
CTDT	7	8	4	100
CTDD	10	2	1	300
Conjoint Triad descriptor related				
CTriad	10	6	1	200
KSCTriad	9	4	1	300
Sequence order descriptor related				
SOCNumber	10	4	3	100
QSOrder	9	6	1	400
D-Script related				
D-script	9	6	1	200
ESM related				
ESM	10	4	1	300

Table B. Optimized hyper-parameters for the gradient boosting classification models with different features.

Hyper-parameters for the gradient boosting classification models				
Feature	max_depth	min_samples_split	min_samples_leaf	n_estimators
Amino acid composition related				
AAC	9	4	1	100
CKSAAP	5	2	1	400
TPC	8	10	1	200
DPC	8	10	3	100
DDE	6	8	2	400
PAAC	10	10	1	300
APAAC	8	10	1	400
Grouped amino acid composition related				
GAAC	10	10	3	200
CKSAAGP	7	8	2	300
GDPC	5	2	1	400
GTPC	7	8	3	200
Distribution of amino acid properties related				
Moran	5	4	2	200
Geary	9	4	3	400
NMBroto	8	6	3	100
Amino acid distribution patterns related				
CTDC	9	6	1	400
CTDT	10	10	1	300
CTDD	7	10	1	400
Conjoint Triad descriptor related				
CTriad	4	2	1	400
KSCTriad	5	2	1	400
Sequence order descriptor related				
SOCNumber	8	6	1	400
QSOrder	7	6	1	200
D-Script related				
D-script	7	8	2	300
ESM related				
ESM	5	4	1	100

2 Performance of random forest and gradient boosting classification models with different features on the training set

With the tuned hyper-parameters, Random Forest and gradient boosting classification models corresponding to 21 Ifeature descriptors, D-script contact feature and ESM feature were evaludated by the 5-fold cross validation method on the training set. Average ROC-AUC scores for total 42 classification models were shown in Fig A.

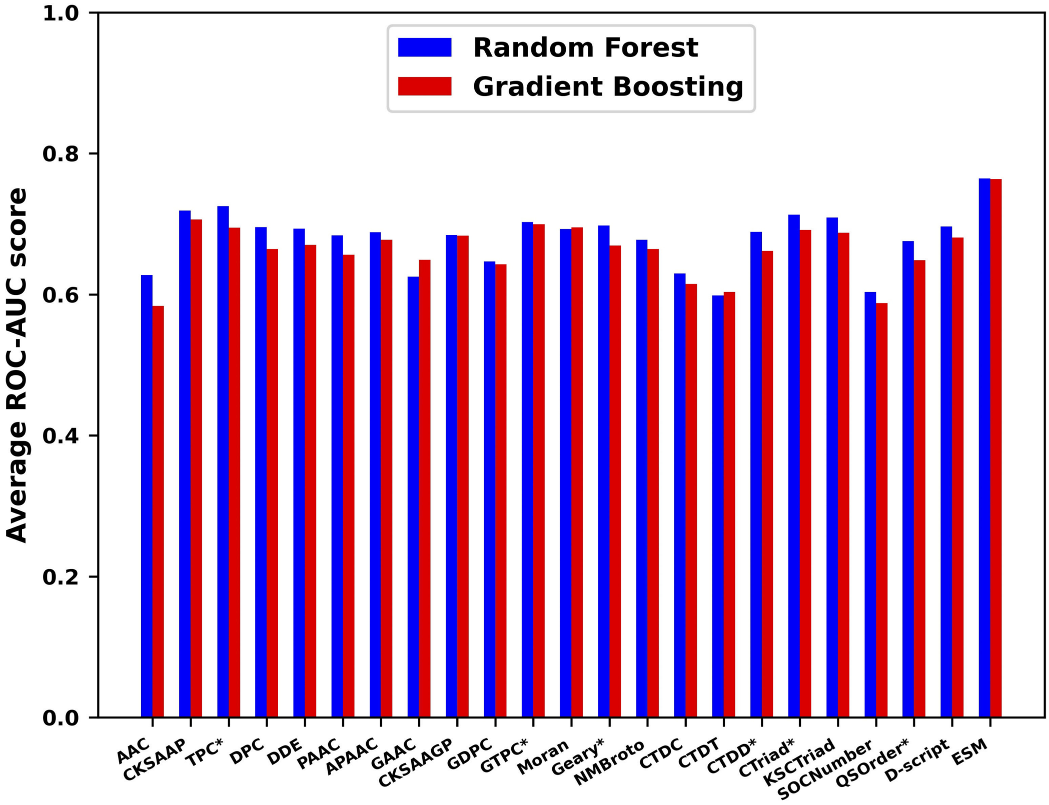

Fig A. Average ROC-AUC scores for random forest and gradient boosting classification models with different features.

For the models based on Ifeature, only the best ones in each group were selected here and compared with D-script contact feature and ESM feature, including TPC, GTPC, Geary, CTDD, CTriad and QSOrder.

ROC-AUC curve, fpr-threshold curve and precision-recall curve for the random forest classification models with different features were shown in Figs B-D, separately. ROC-AUC curve, fpr-threshold curve and precision-recall curve for the gradient boosting classification models with different features were shown in Figs E-G, separately.

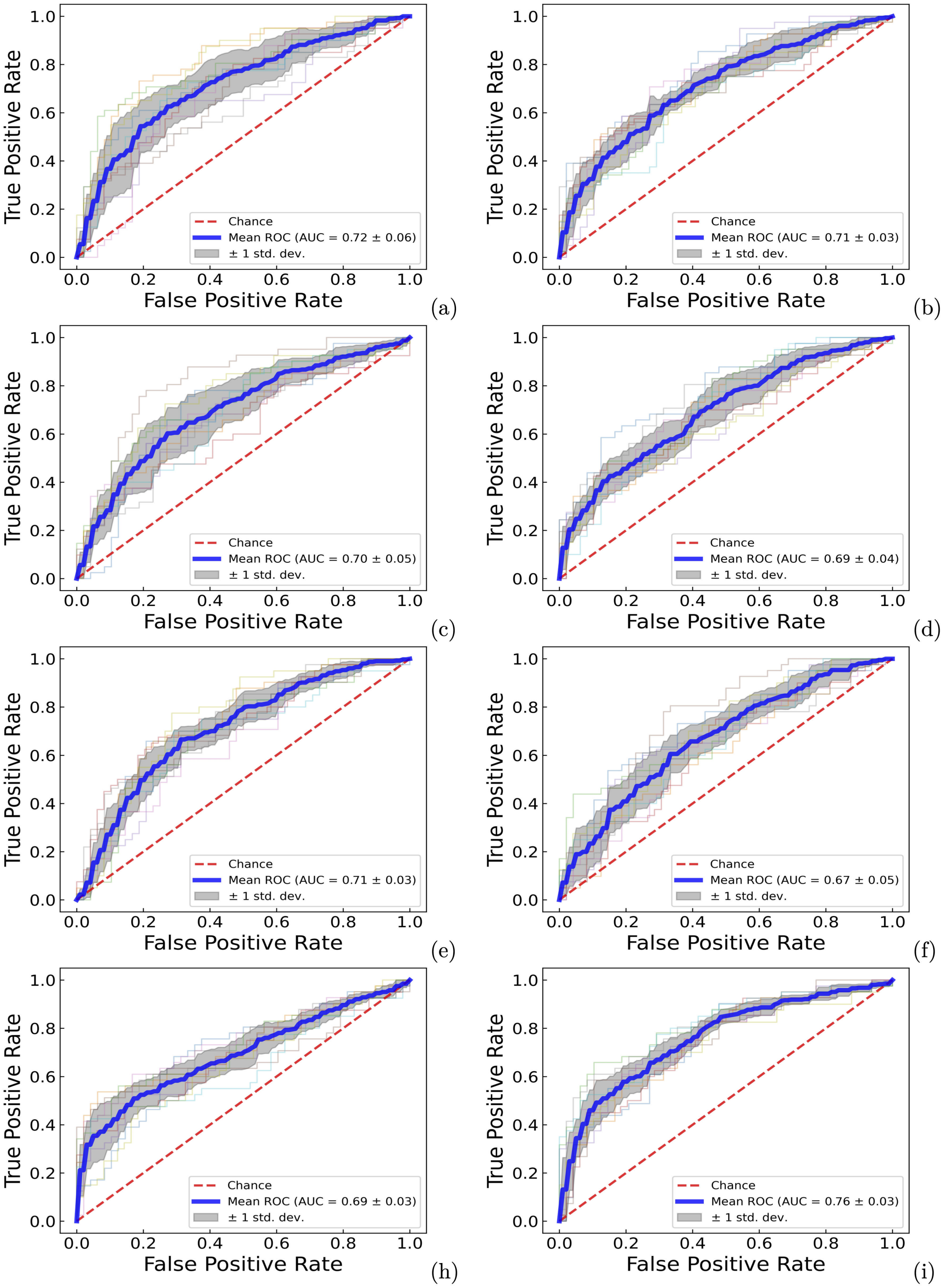

Fig B. ROC-AUC curves for random forest classification models with different features. (a) TPC. (b) GTPC. (c) Geary. (d) CTDD. (e) CTriad. (f) QSOrder. (h) D-script. (i) ESM.

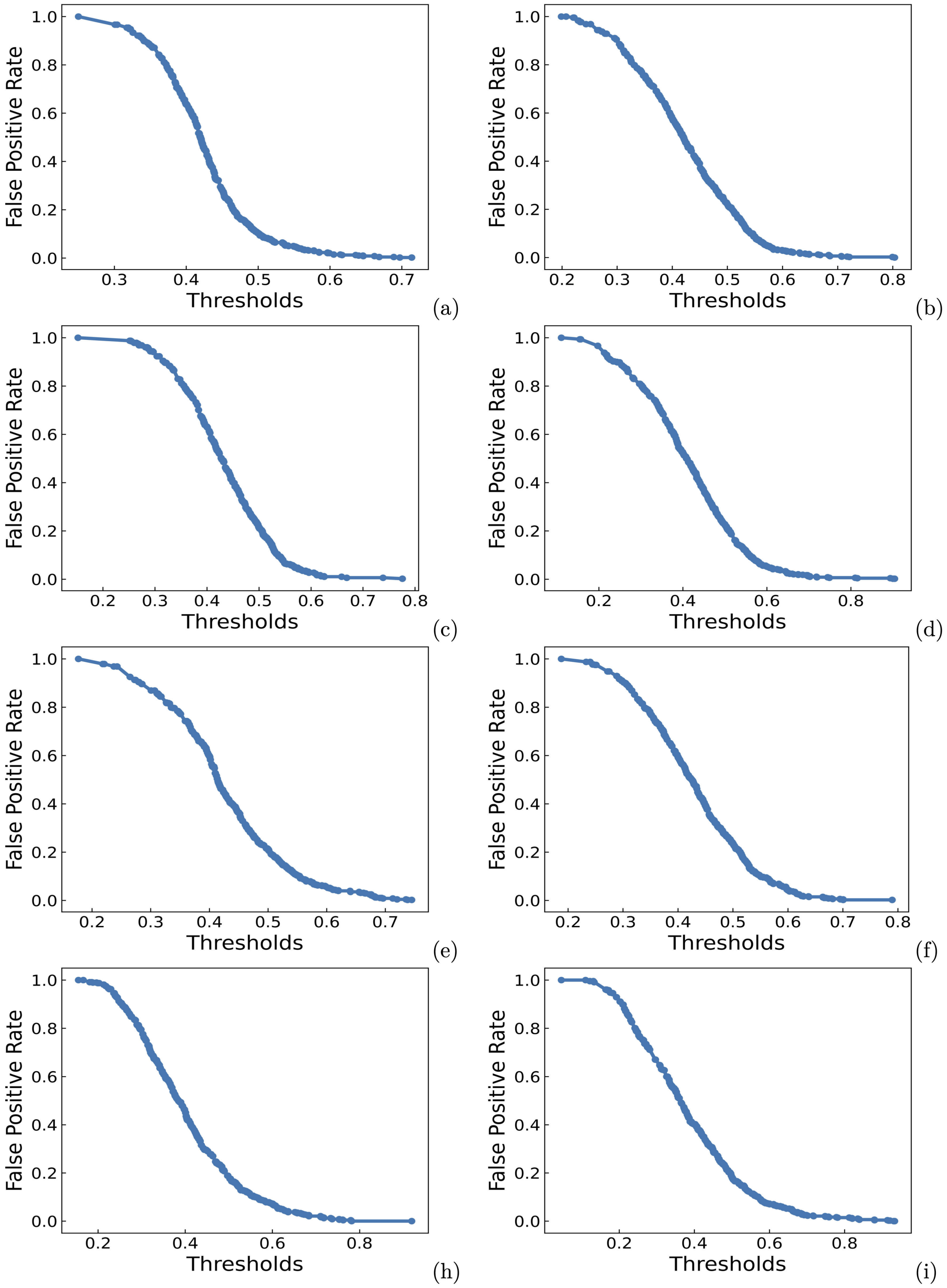

Fig C. False positive rate - threshold curves for random forest classification models with different features. (a) TPC. (b) GTPC. (c) Geary. (d) CTDD. (e) CTriad. (f) QSOrder. (h) D-script. (i) ESM.

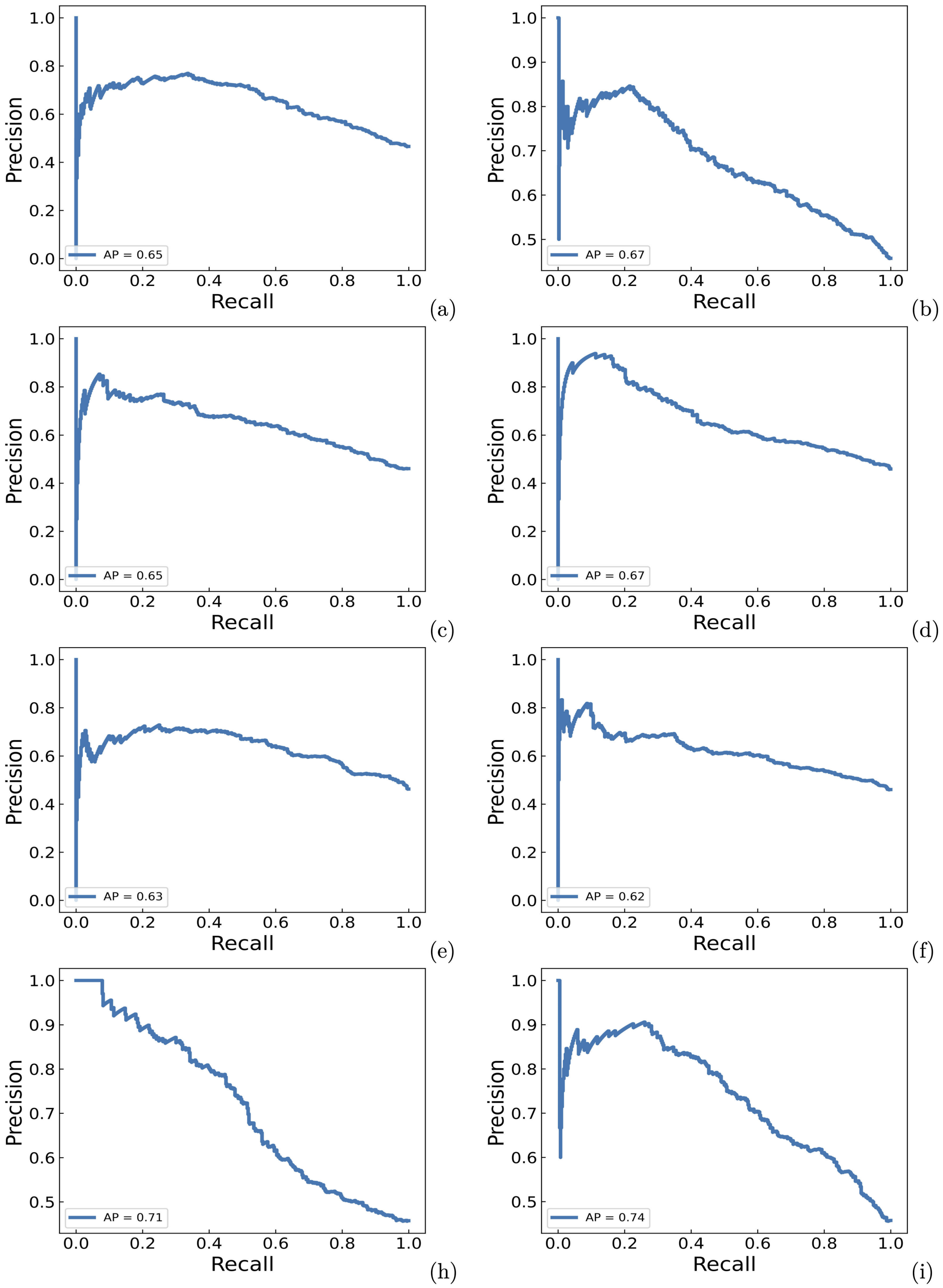

Fig D. Precision - recall curves for random forest classification models with different features. (a) TPC. (b) GTPC. (c) Geary. (d) CTDD. (e) CTriad. (f) QSOrder. (h) D-script. (i) ESM.

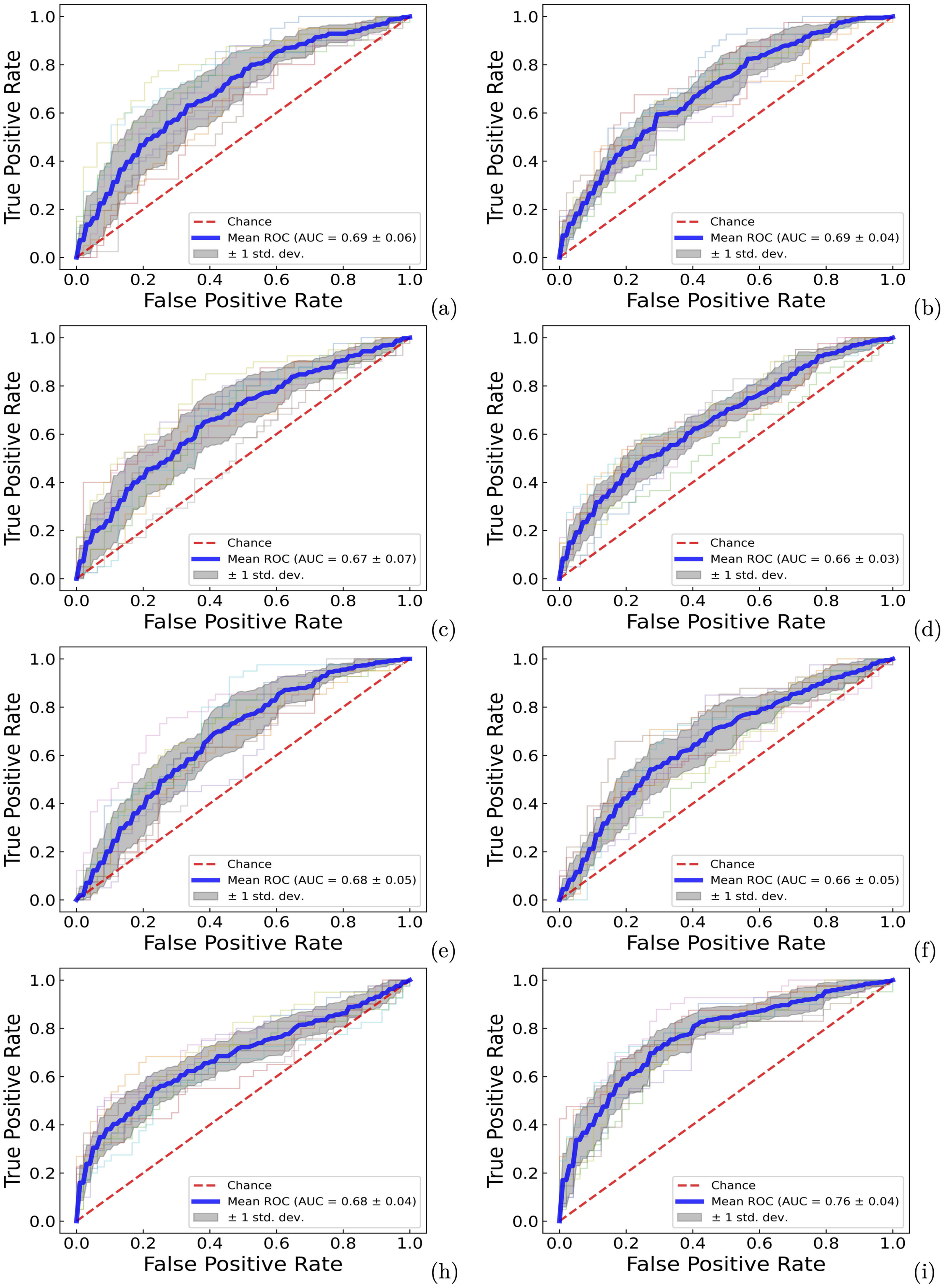

Fig E. ROC-AUC curves for the gradient boosting classification models with different features. (a) TPC. (b) GTPC. (c) Geary. (d) CTDD. (e) CTriad. (f) QSOrder. (h) D-script. (i) ESM.

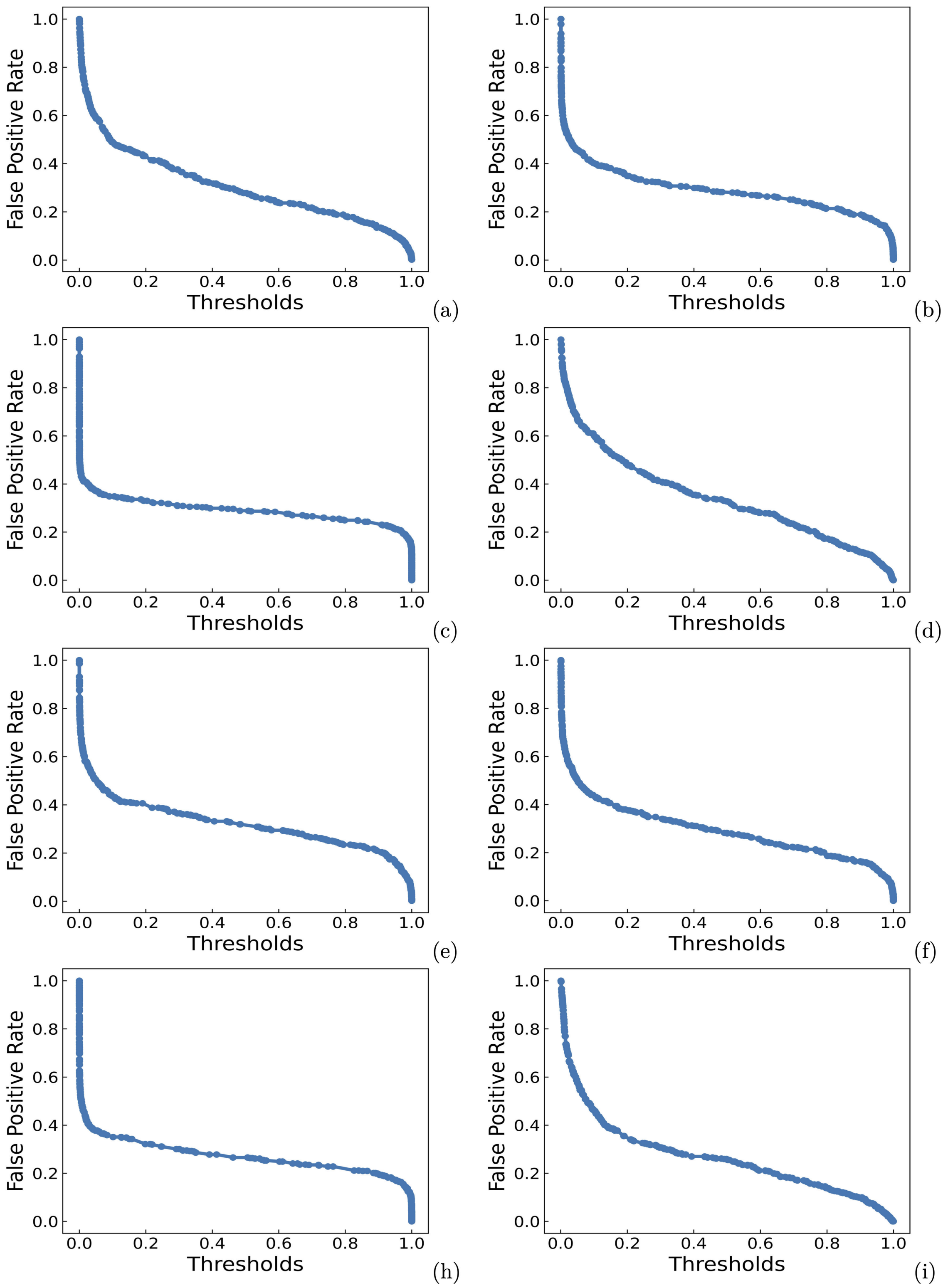

Fig F. False positive rate - threshold curves for the gradient boosting classification models with different features. (a) TPC. (b) GTPC. (c) Geary. (d) CTDD. (e) CTriad. (f) QSOrder. (h) D-script. (i) ESM.

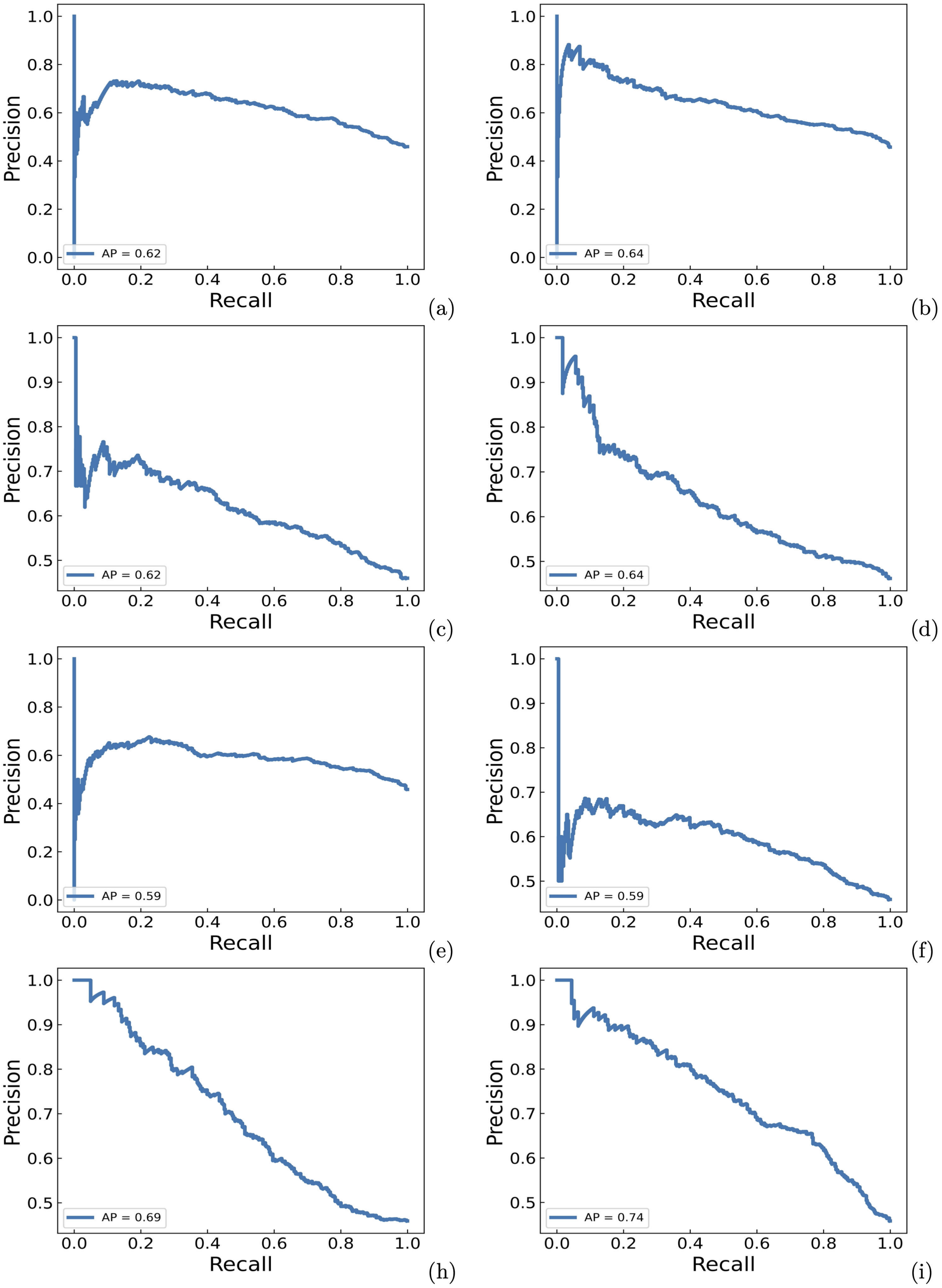

Fig G. Precision - recall curves for the gradient boosting classification models with different features. (a) TPC. (b) GTPC. (c) Geary. (d) CTDD. (e) CTriad. (f) QSOrder. (h) D-script. (i) ESM.

2.1 Performance of RF model with combined features on the training set

In order to investigate whether the performance could be improved when adding other features with ESM feature, TPC, GTPC, Geary, CTDD, CTriad, QSOrder and Dscript features were combined with ESM feature. Average ROC-AUC scores and average precision scores were shown in Fig H.

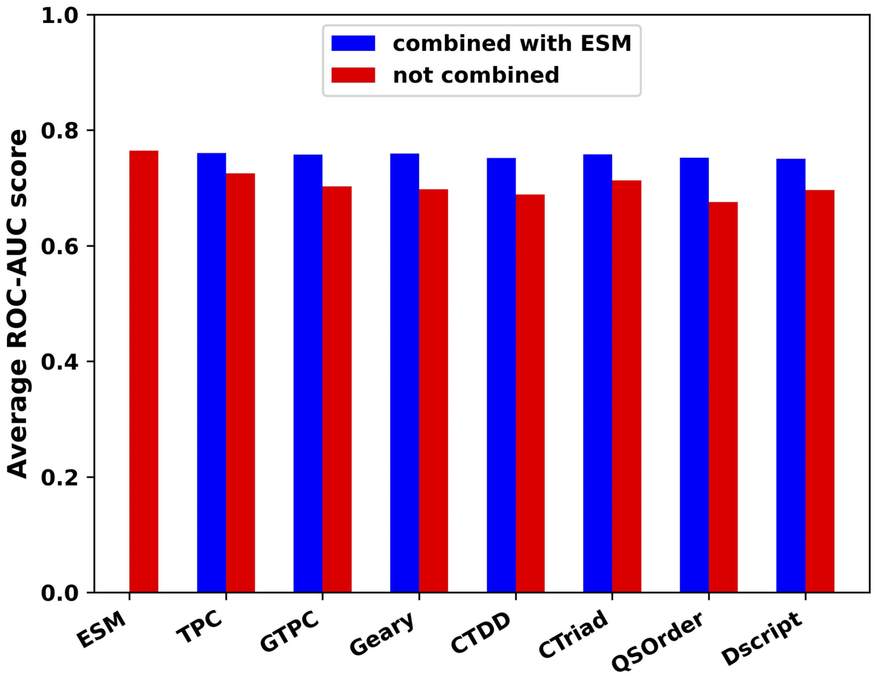

(a)

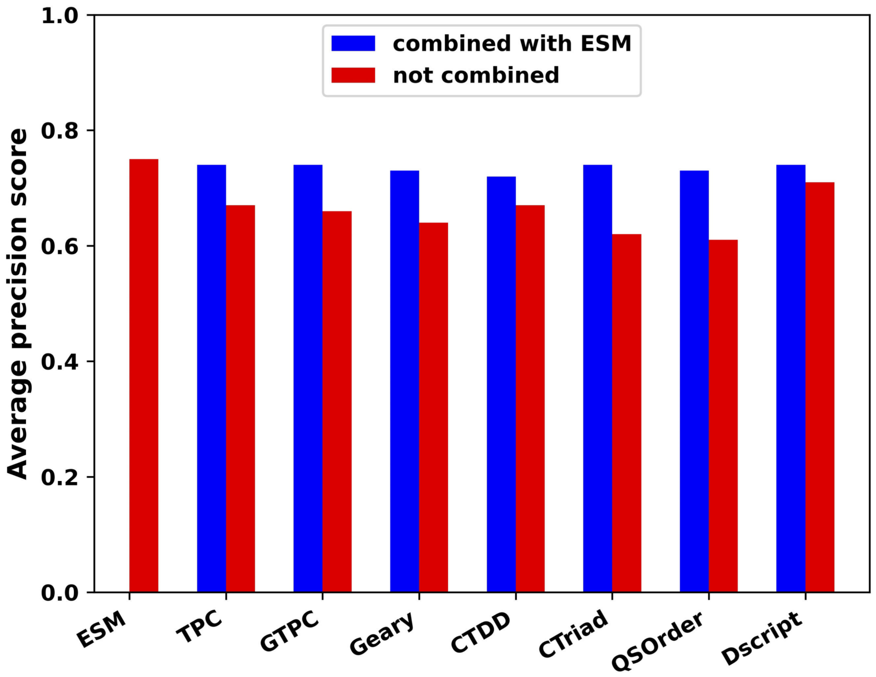

(b)

Fig H. Average ROC-AUC scores and precision scores for random forest models with combined features. (a) Average ROC-AUC score. (b) Average precision score.

2.2 Performance of RF and GBT ensemble models

In order to investigate whether the performance could be improved when combining the RF and GBT models, two different ensemble methods were used: one is the soft voting method and the other one is the consensus method. Average ROC-AUC, false positive rate-threshold, precision-recall curves for them on the training set were shown in Fig I. Average ROC-AUC, false positive rate-threshold, precision-recall curves of these models on the external test set were shown in Fig J.

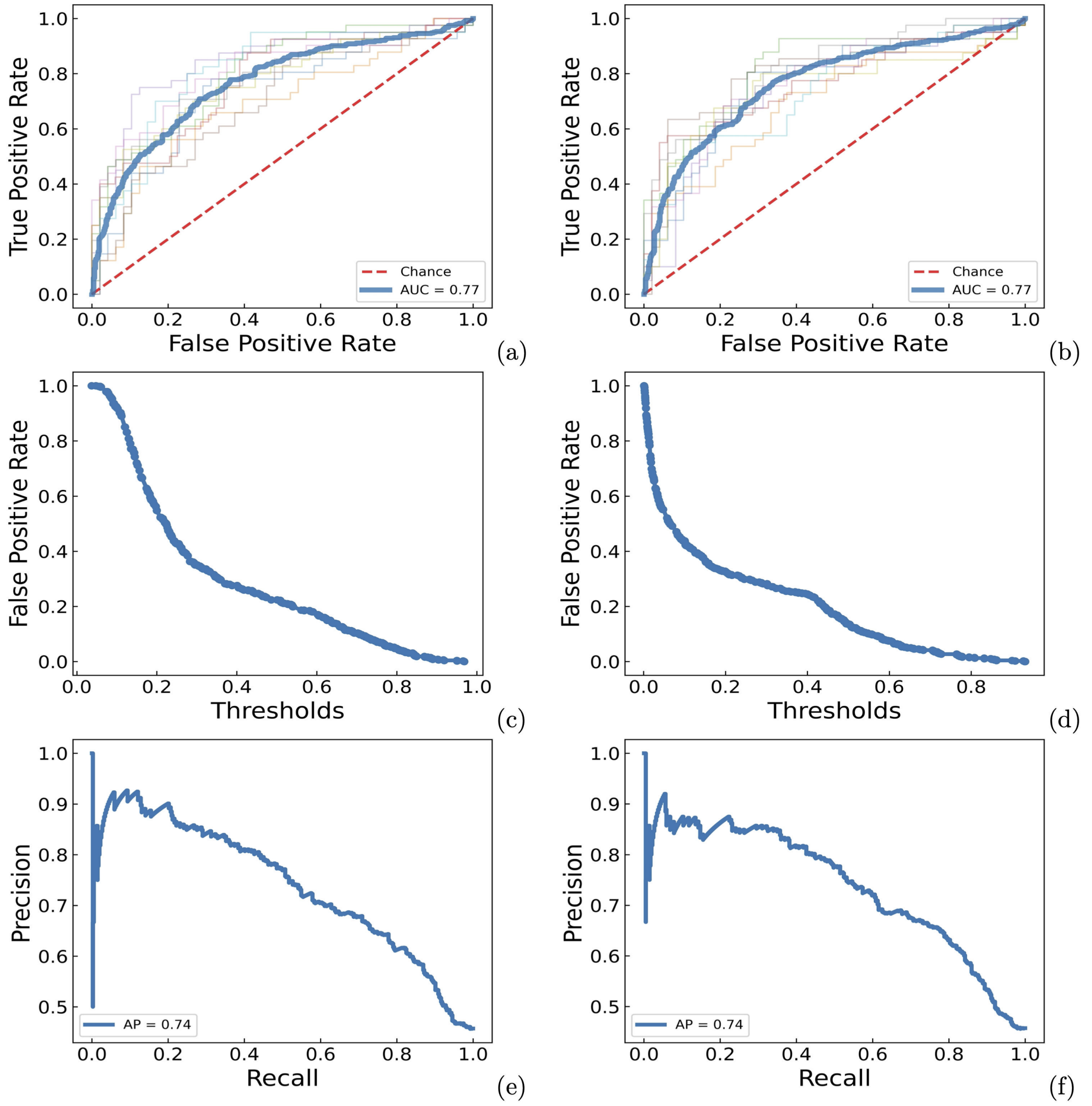

Fig I. ROC-AUC, false positive rate-threshold, precision-recall curves for the soft voting model and consensus model on the training set. (a) ROC-AUC curve for soft voting model. (b) ROC-AUC curve for consensus model. (c) false positive rate-threshold curve for soft voting model. (d) false positive rate-threshold curve for consensus model. (e) precision-recall curve for soft voting model. (f) precision-recall curve for consensus model.

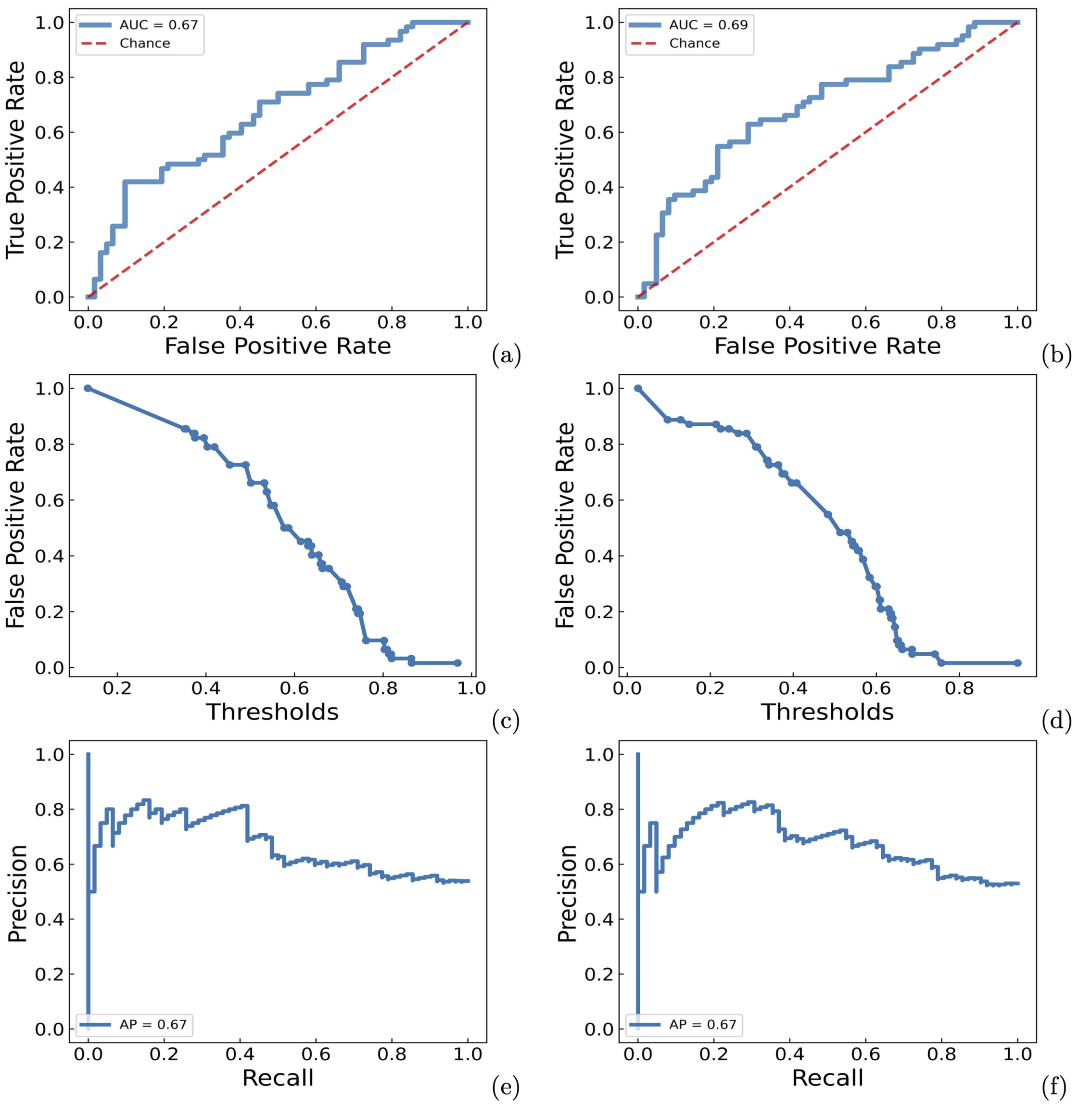

Fig J. ROC-AUC, false positive rate-threshold, precision-recall curves for the soft voting model and consensus model on the external test set. (a) ROC-AUC curve for soft voting model. (b) ROC-AUC curve for consensus model. (c) false positive rate-threshold curve for soft voting model. (d) false positive rate-threshold curve for consensus model. (e) precision-recall curve for soft voting model. (f) precision-recall curve for consensus model.

2.3 Sequence similarity between the proteins in the training set and external test set

For each protein in the test set, sequence alignments with BlastP [1] was applied to search against all the proteins in the training set and the smallest E-value was selected to represent the relationship between this protein and the protein kinases in the training set. Distribution of the smallest E-value for the protein in the external test set was shown in Fig K.

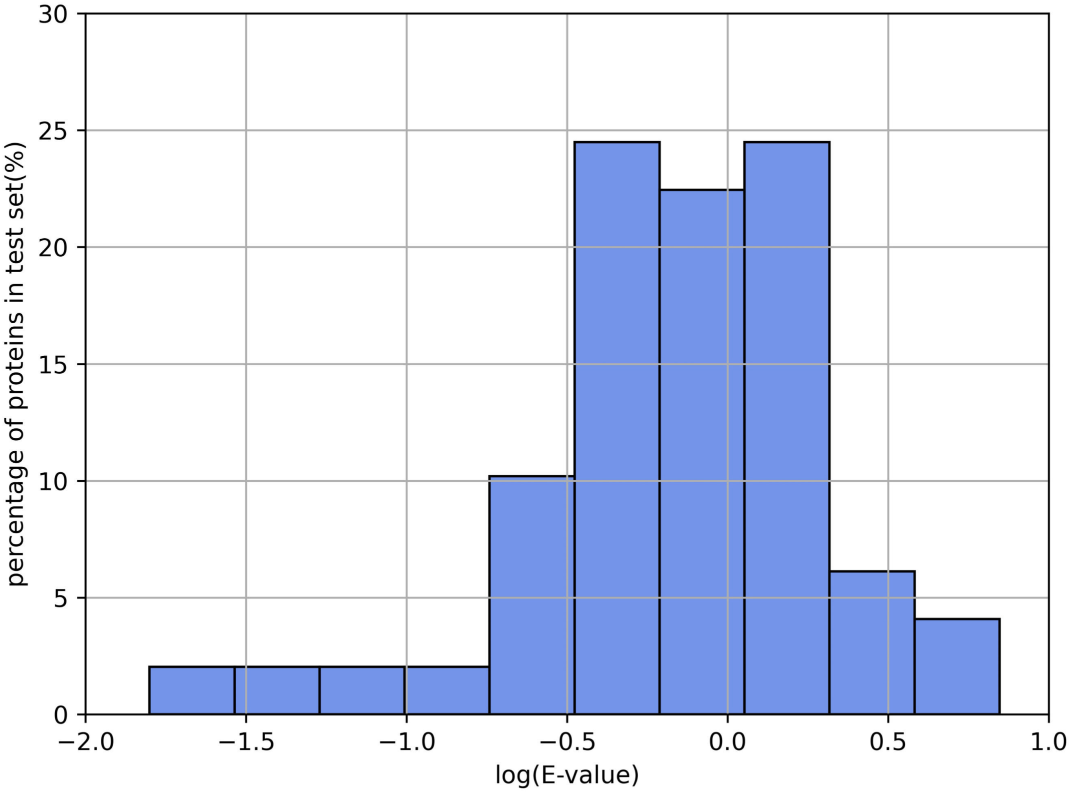

Fig K. Distribution of logarithm of the smallest E-values for proteins in the external test set.

2.4 Performance of RF model on the second test set

Average ROC-AUC, false positive rate-threshold, precision-recall curves of the RF model on the second test set were shown in Fig L.

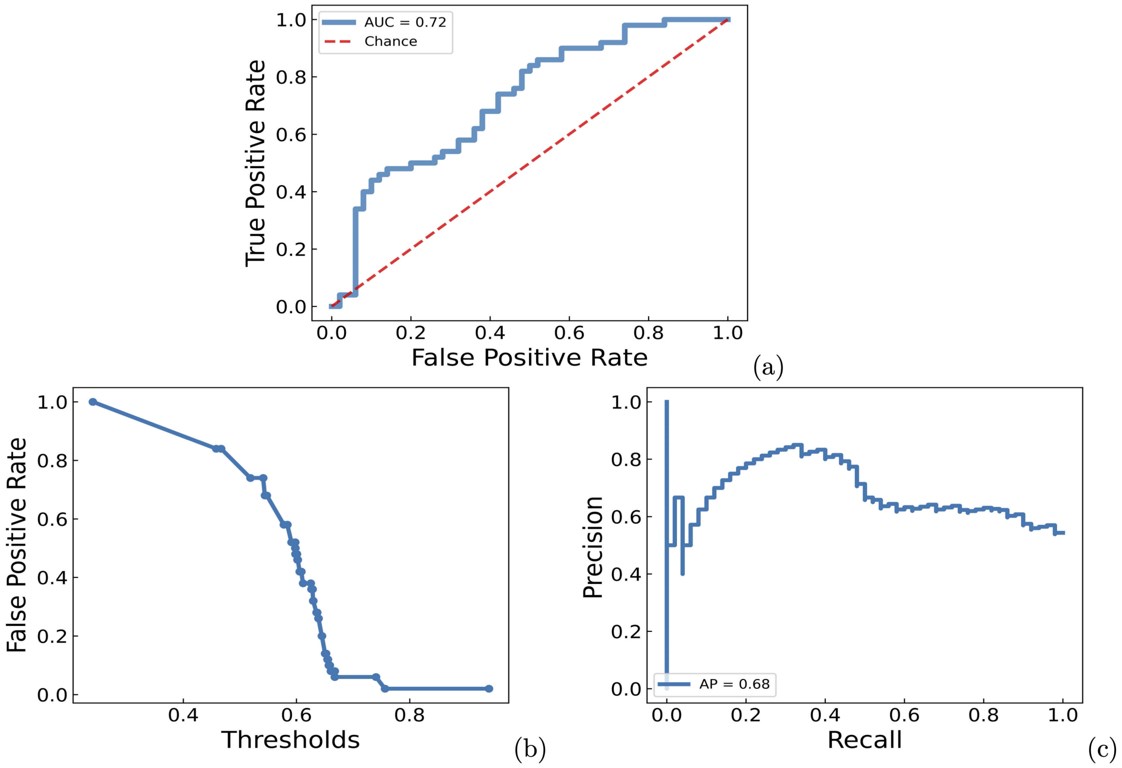

Fig L. ROC-AUC, false positive rate-threshold, precision-recall curves for the RF models on the external test set in which the negative samples are not in the same superfamilies with the positive proteins. (a) ROC-AUC curve. (b) False positive rate-threshold curve . (c) Precision-recall curve. AUC represents the ROC-AUC score, AP represents the average precision score.

2.5 eSHAP analysis for the protein kinases

The structural mapping of the key positions and key-position only MSA for the protein kinases were shown in Figs M-X.

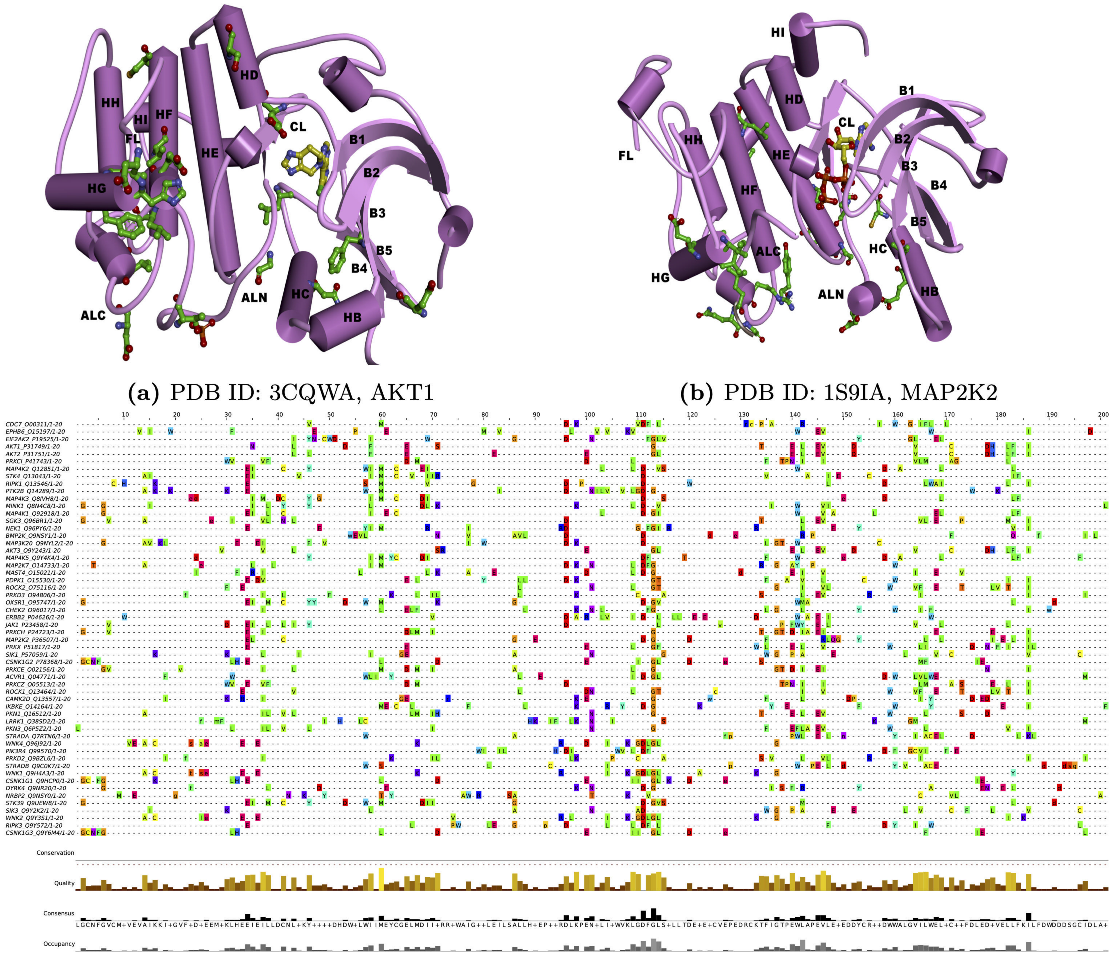

(a) PDB ID: 3CQWA, AKT1

(b) PDB ID: 1S9IA, MAP2K2

(c) multiple sequence alignments of the top ranked positions

Fig M. Structural mapping of the top ranked positions for positive and negative samples in protein kinase subgroup 1 and multiple sequence alignments of the top ranked positions in this subgroup. Purple represents the structure of protein kinase; yellow balls and sticks represent co-crystallized ligand; green balls and sticks represent the predicted key positions.

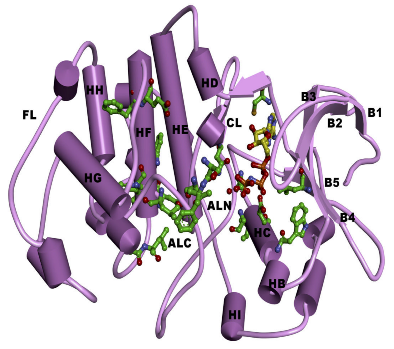

(a) PDB ID: 4IC7D, MAPK7

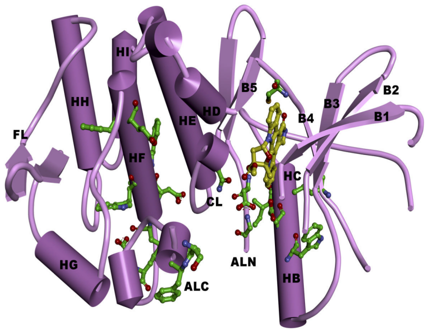

(b) PDB ID: 4KIKA, IKBKB

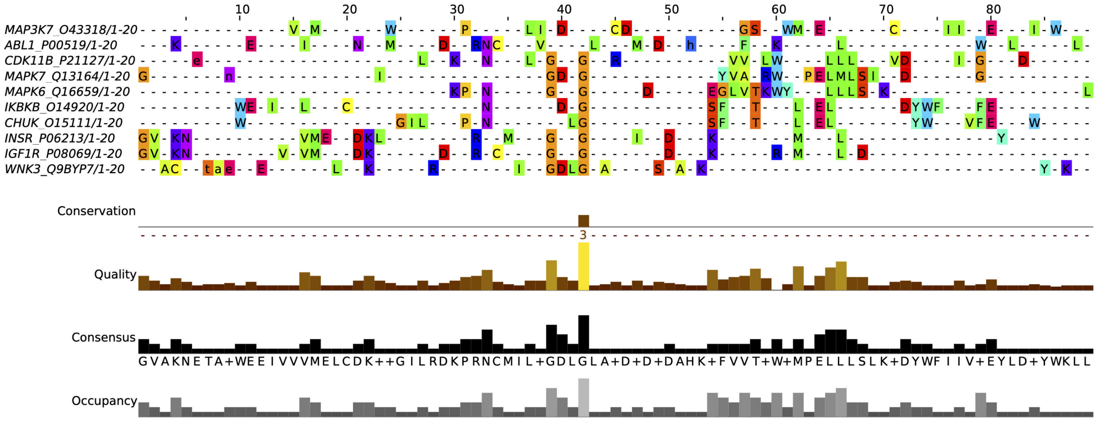

(c) multiple sequence alignments of the top ranked positions

Fig N. Structural mapping of the top ranked positions for positive and negative samples in protein kinase subgroup 2 and multiple sequence alignments of the top ranked positions in this subgroup. Purple represents the structure of protein kinase; yellow balls and sticks represent co-crystallized ligand; green balls and sticks represent the predicted key positions.

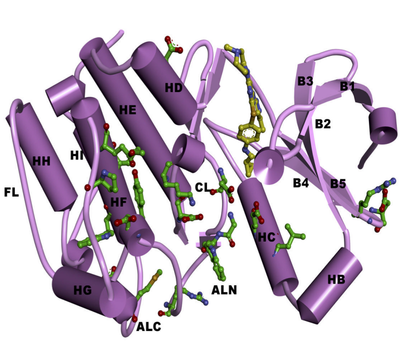

(a) PDB ID: 3E5A, AURKA

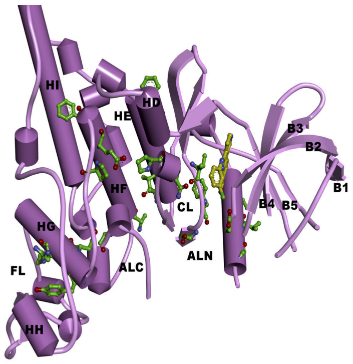

(b) PDB ID: 6P5SA, HIPK2

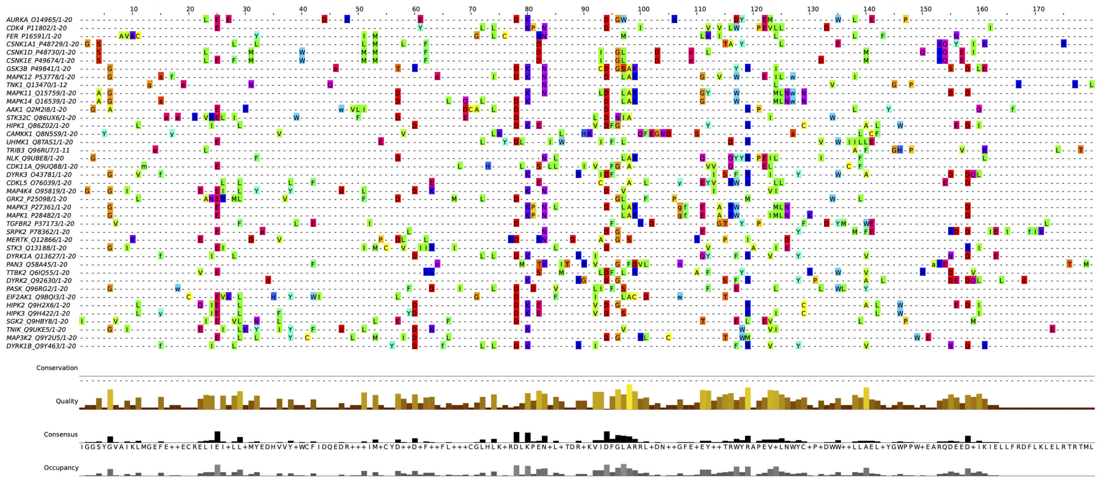

(c) multiple sequence alignments of the top ranked positions

Fig O. Structural mapping of the top ranked positions for positive and negative samples in protein kinase subgroup 3 and multiple sequence alignments of the top ranked positions in this subgroup. Purple represents the structure of protein kinase; yellow balls and sticks represent co-crystallized ligand; green balls and sticks represent the predicted key positions.

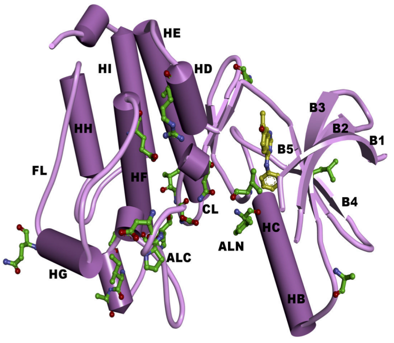

(a) PDB ID: 5L6OA, EPHB3

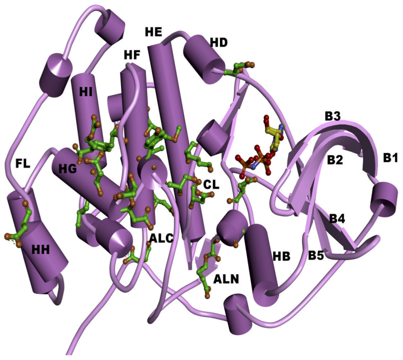

(b) PDB ID: 2PVRA, CSNK2A1

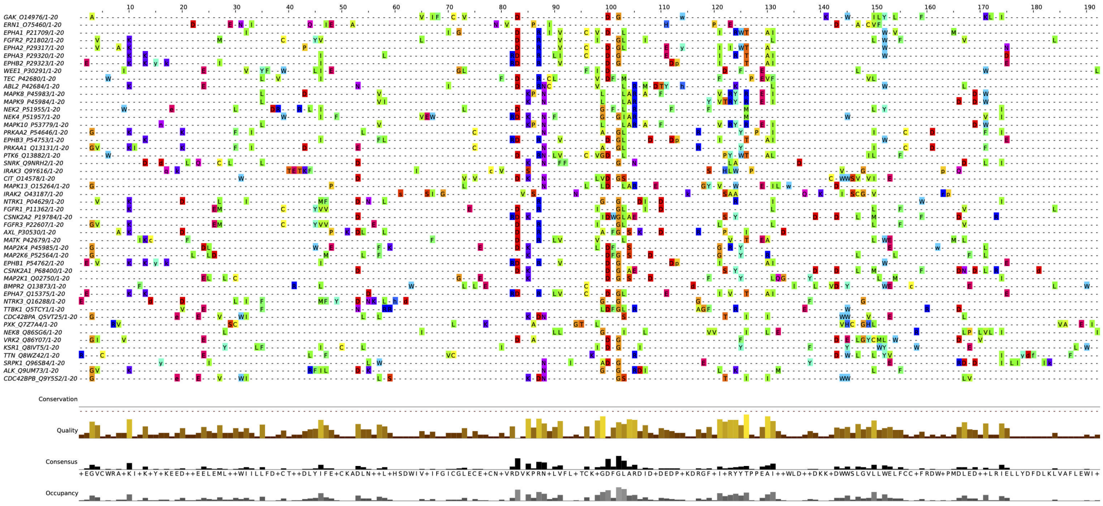

(c) multiple sequence alignments of the top ranked positions

Fig P. Structural mapping of the top ranked positions for positive and negative samples in protein kinase subgroup 4 and multiple sequence alignments of the top ranked positions in this subgroup. Purple represents the structure of protein kinase; yellow balls and sticks represent co-crystallized ligand; green balls and sticks represent the predicted key positions.

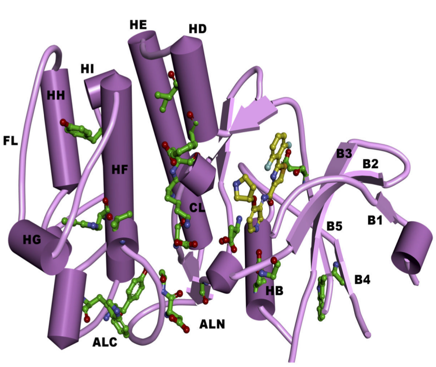

(a) PDB ID: 4X7QB, PIM2

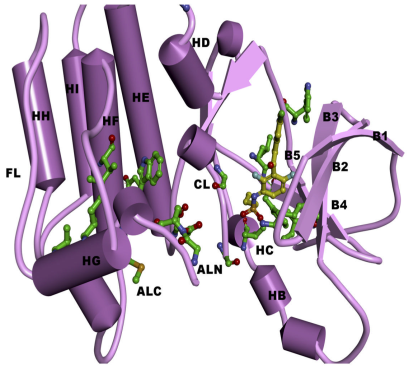

(b) PDB ID: 3C4CA, BRAF

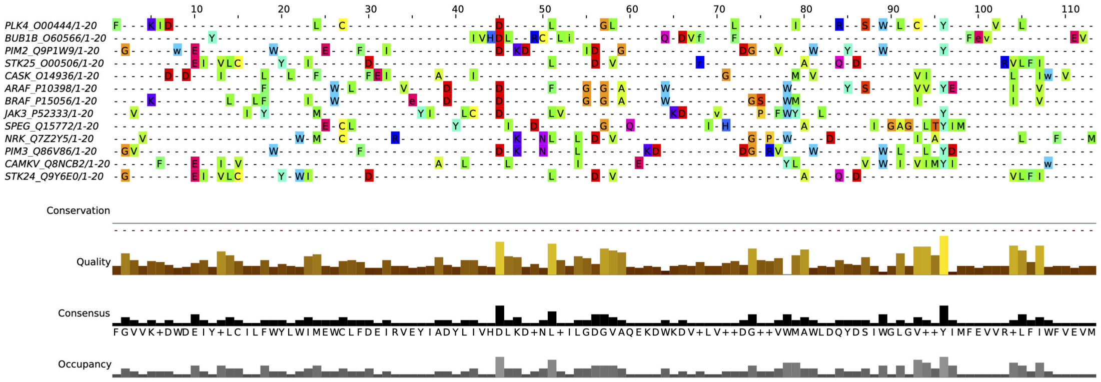

(c) multiple sequence alignments of the top ranked positions

Fig Q. Structural mapping of the top ranked positions for positive and negative samples in protein kinase subgroup 5 and multiple sequence alignments of the top ranked positions in this subgroup. Purple represents the structure of protein kinase; yellow balls and sticks represent co-crystallized ligand; green balls and sticks represent the predicted key positions.

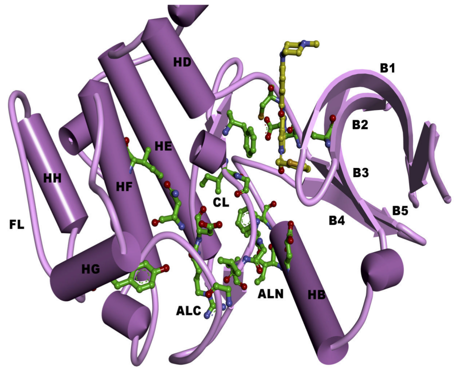

(a) PDB ID: 2OWB, PLK1

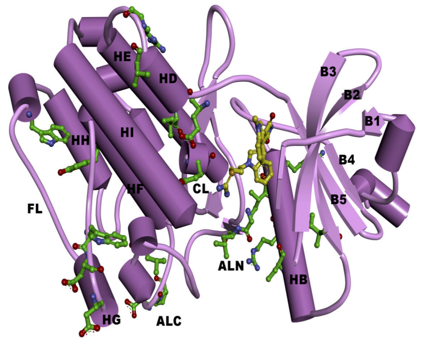

(b) PDB ID: 2V7OA, CAMK

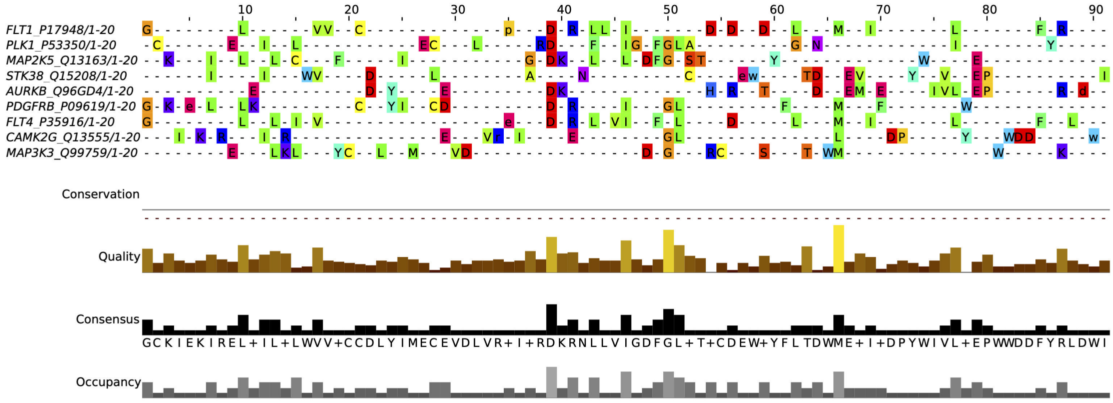

(c) multiple sequence alignments of the top ranked positions

Fig R. Structural mapping of the top ranked positions for positive and negative samples in protein kinase subgroup 6 and multiple sequence alignments of the top ranked positions in this subgroup. Purple represents the structure of protein kinase; yellow balls and sticks represent co-crystallized ligand; green balls and sticks represent the predicted key positions.

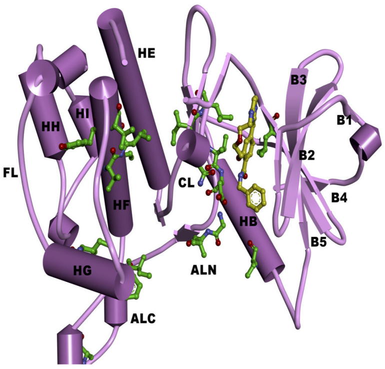

(a) PDB ID: 7QUEA, STK17A

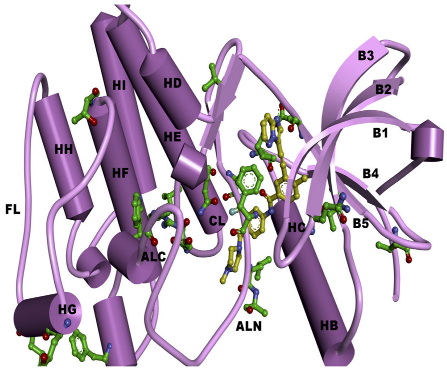

(b) PDB ID: 4QRCA, FGFR4

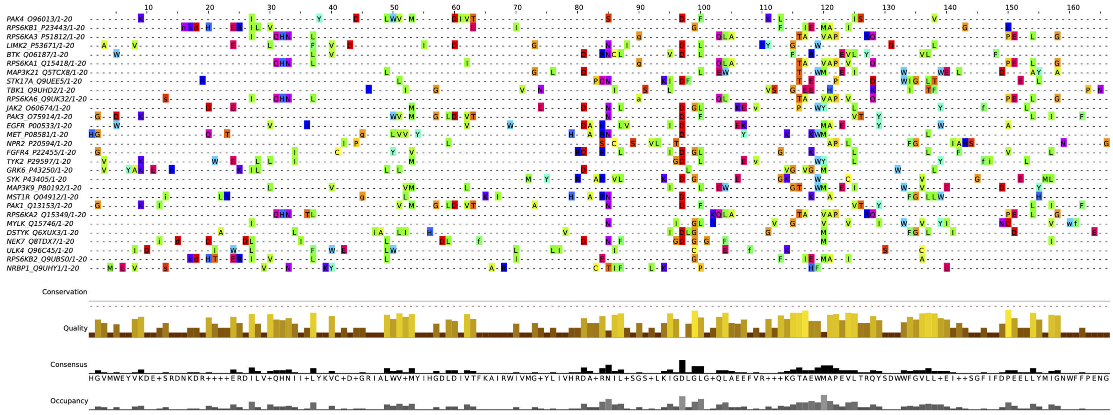

(c) multiple sequence alignments of the top ranked positions

Fig S. Structural mapping of the top ranked positions for positive and negative samples in protein kinase subgroup 7 and multiple sequence alignments of the top ranked positions in this subgroup. Purple represents the structure of protein kinase; yellow balls and sticks represent co-crystallized ligand; green balls and sticks represent the predicted key positions.

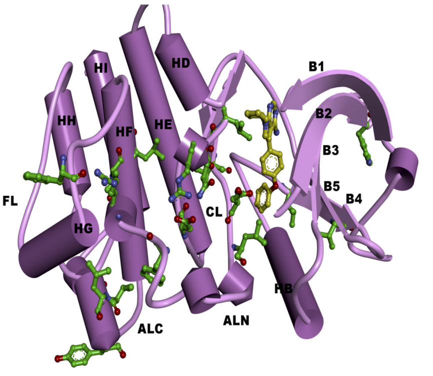

(a) PDB ID: 7QHGA, LIMK2

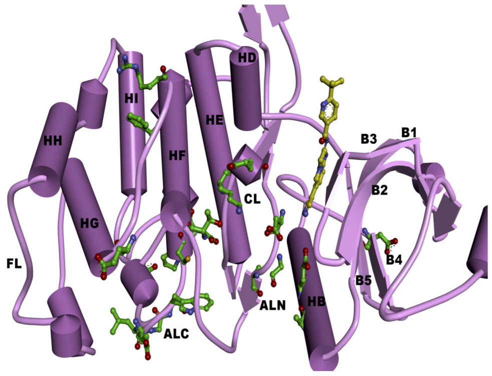

(b) PDB ID: 6FYRA, CLK3

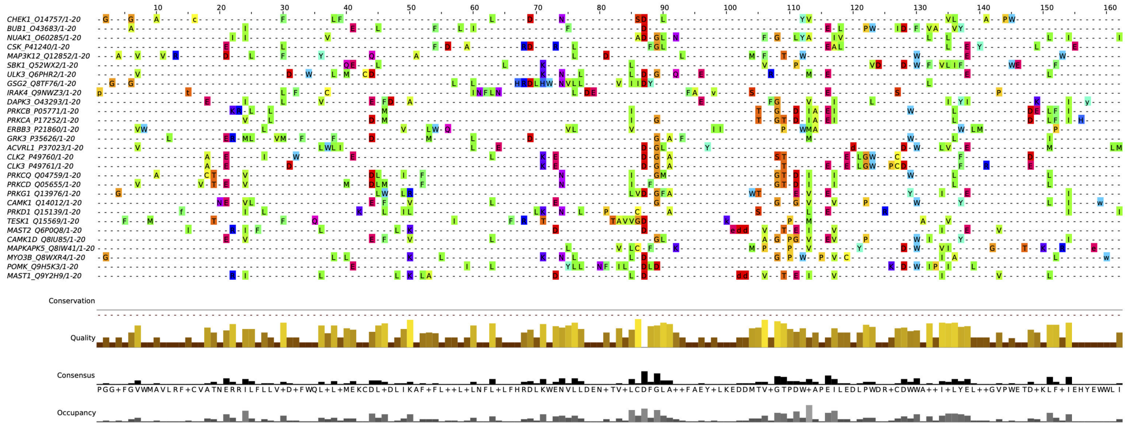

(c) multiple sequence alignments of the top ranked positions

Fig T. Structural mapping of the top ranked positions for positive and negative samples in protein kinase subgroup 8 and multiple sequence alignments of the top ranked positions in this subgroup. Purple represents the structure of protein kinase; yellow balls and sticks represent co-crystallized ligand; green balls and sticks represent the predicted key positions.

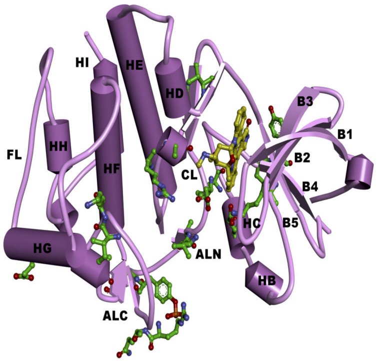

(a) PDB ID: 2DQ7X, FYN

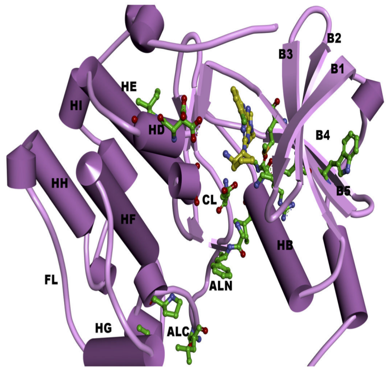

(b) PDB ID: 2F57B, PAK5

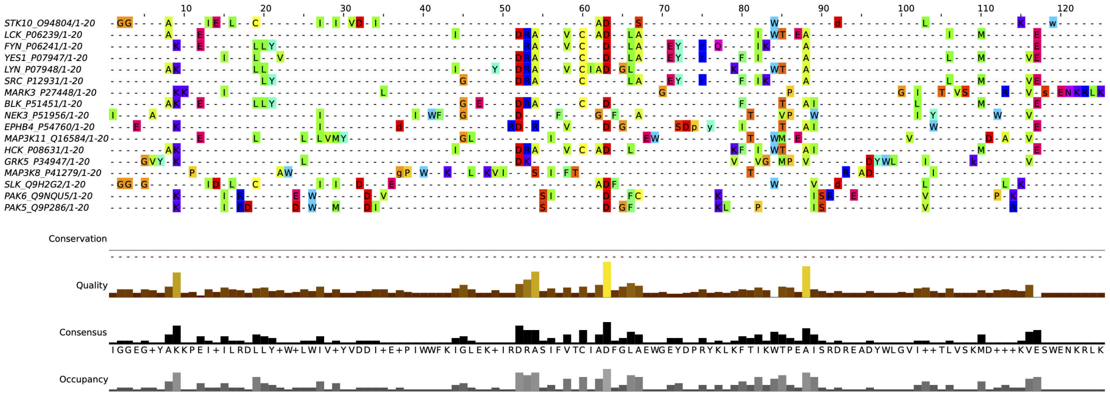

(c) multiple sequence alignments of the top ranked positions

Fig U. Structural mapping of the top ranked positions for positive and negative samples in protein kinase subgroup 9 and multiple sequence alignments of the top ranked positions in this subgroup. Purple represents the structure of protein kinase; yellow balls and sticks represent co-crystallized ligand; green balls and sticks represent the predicted key positions.

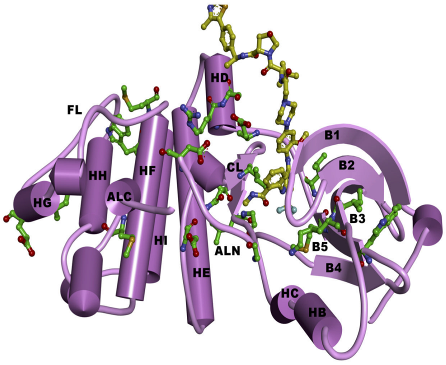

(a) PDB ID: 7PI4D, FAK

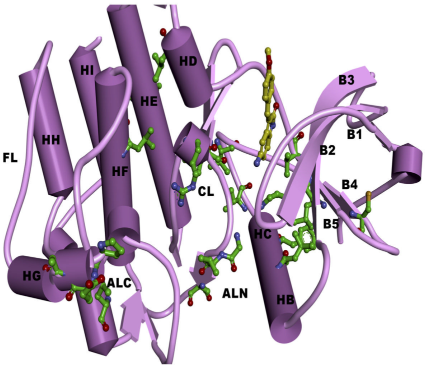

(b) PDB ID: 2X2LA, RET

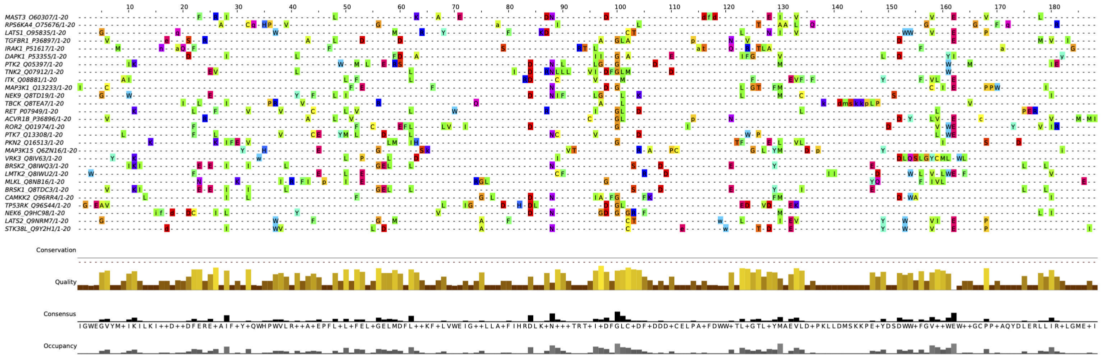

(c) multiple sequence alignments of the top ranked positions

Fig V. Structural mapping of the top ranked positions for positive and negative samples in protein kinase subgroup 10 and multiple sequence alignments of the top ranked positions in this subgroup. Purple represents the structure of protein kinase; yellow balls and sticks represent co-crystallized ligand; green balls and sticks represent the predicted key positions.

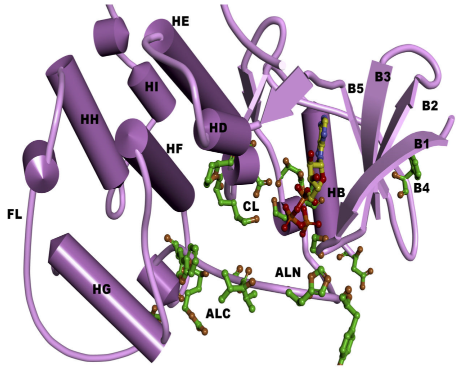

(a) PDB ID: 1UA2A, CDK7

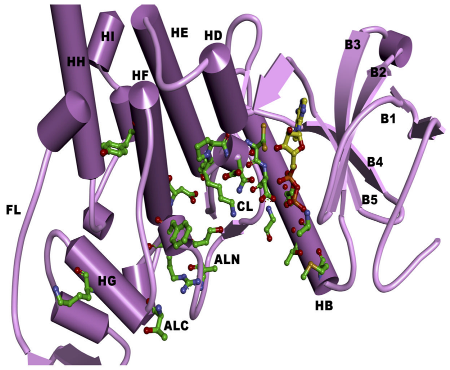

(b) PDB ID: 4IFCA, PRPF4B

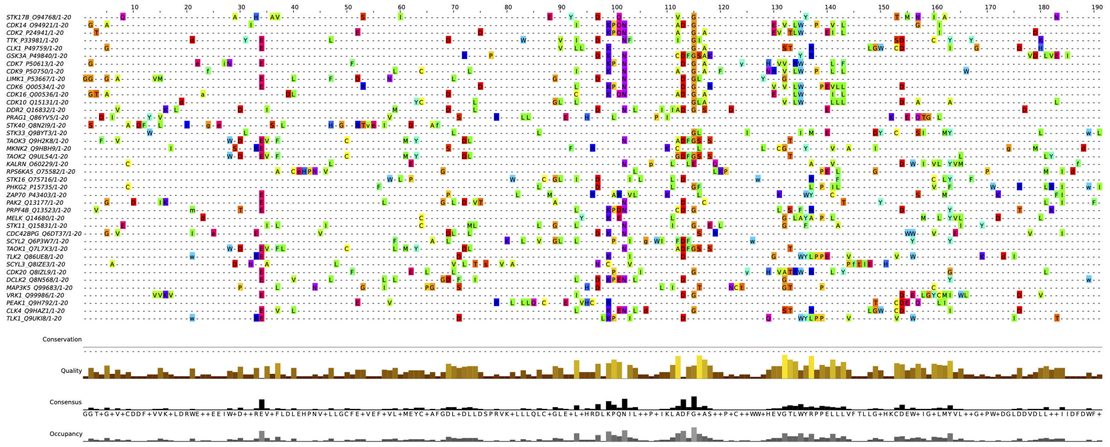

(c) multiple sequence alignments of the top ranked positions

Fig W. Structural mapping of the top ranked positions for positive and negative samples in protein kinase subgroup 11 and multiple sequence alignments of the top ranked positions in this subgroup. Purple represents the structure of protein kinase; yellow balls and sticks represent co-crystallized ligand; green balls and sticks represent the predicted key positions.

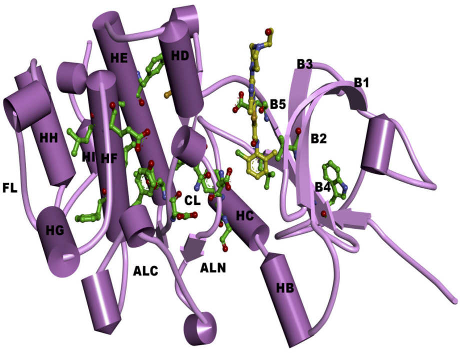

(a) PDB ID: 5VCVA, PKMYT1

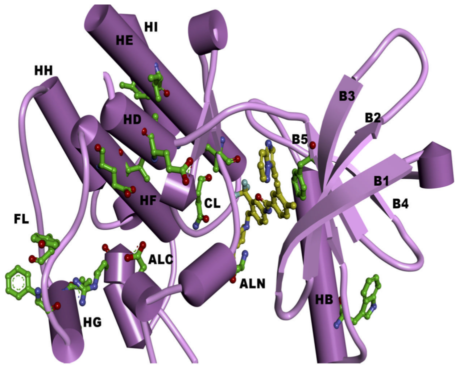

(b) PDB ID: 6TU9A, ROR1

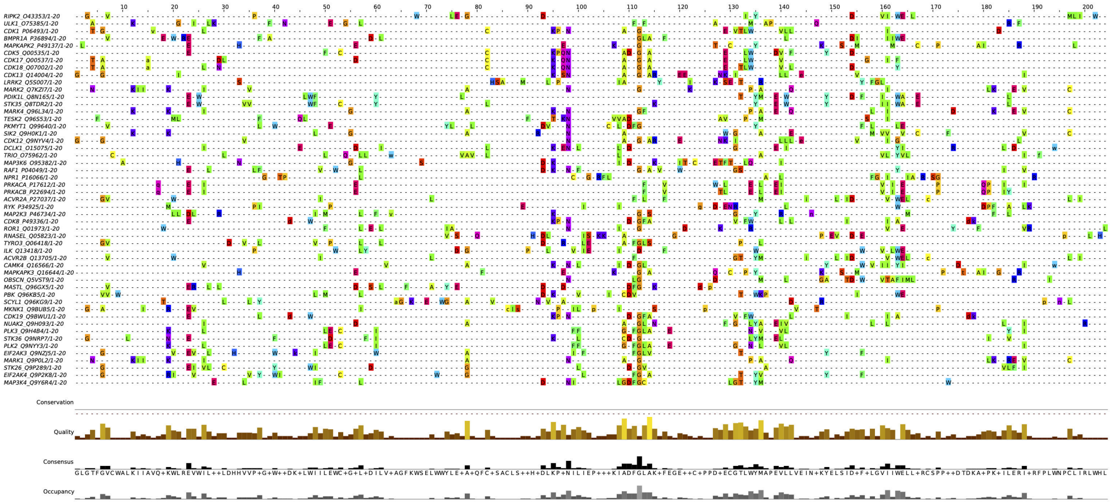

(c) multiple sequence alignments of the top ranked positions

Fig X. Structural mapping of the top ranked positions for positive and negative samples in protein kinase subgroup 12 and multiple sequence alignments of the top ranked positions in this subgroup. Purple represents the structure of protein kinase; yellow balls and sticks represent co-crystallized ligand; green balls and sticks represent the predicted key positions.

2.6 PrePROTAC prediction on human proteins

The probability of whole human proteins to be degraded by CRBN were predicted by the soft voting model and the distribution of probability scores were presented in Fig Y. 615 of them were predicted to be degradable by CRBN with the help of PROTAC binding. Information about these proteins and their predicted probability scores were listed in the supplementary Table C.

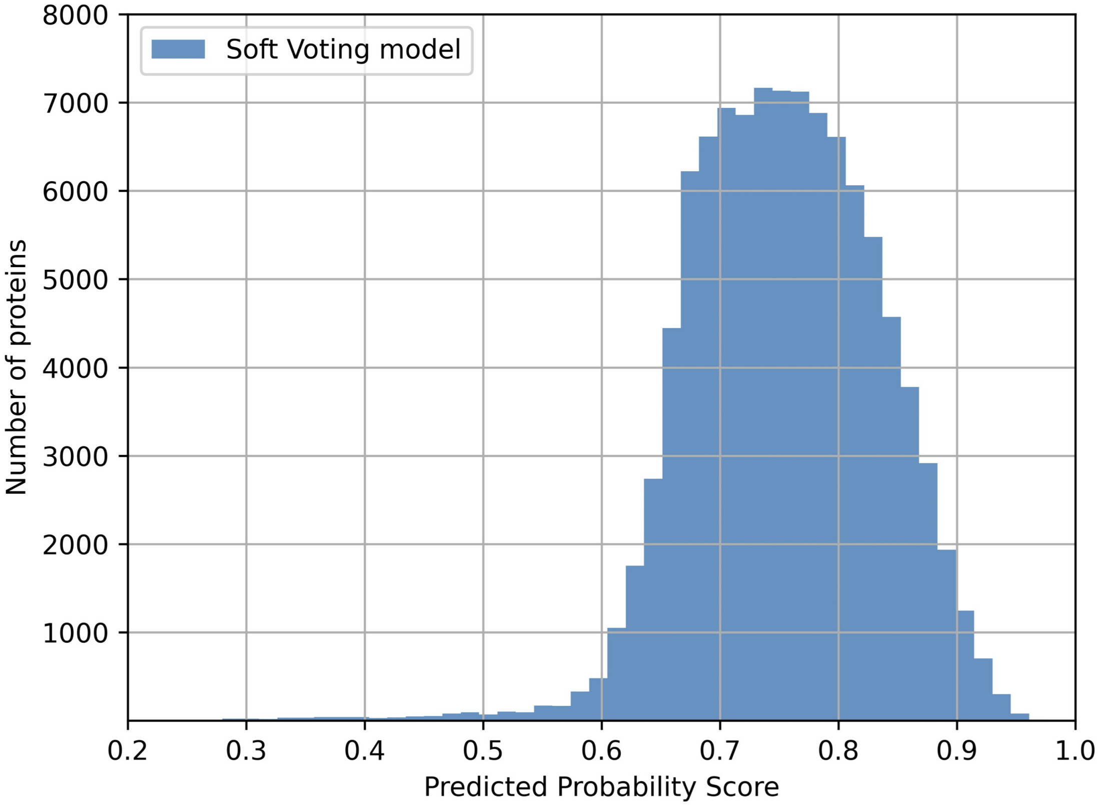

Fig Y. Distribution of predicted probability scores for the whole human proteome.

2.7 Protein kinases in the training data set

Distributions of CRBN and VHL induced degradations in different protein kinase families was shown in Fig Z.

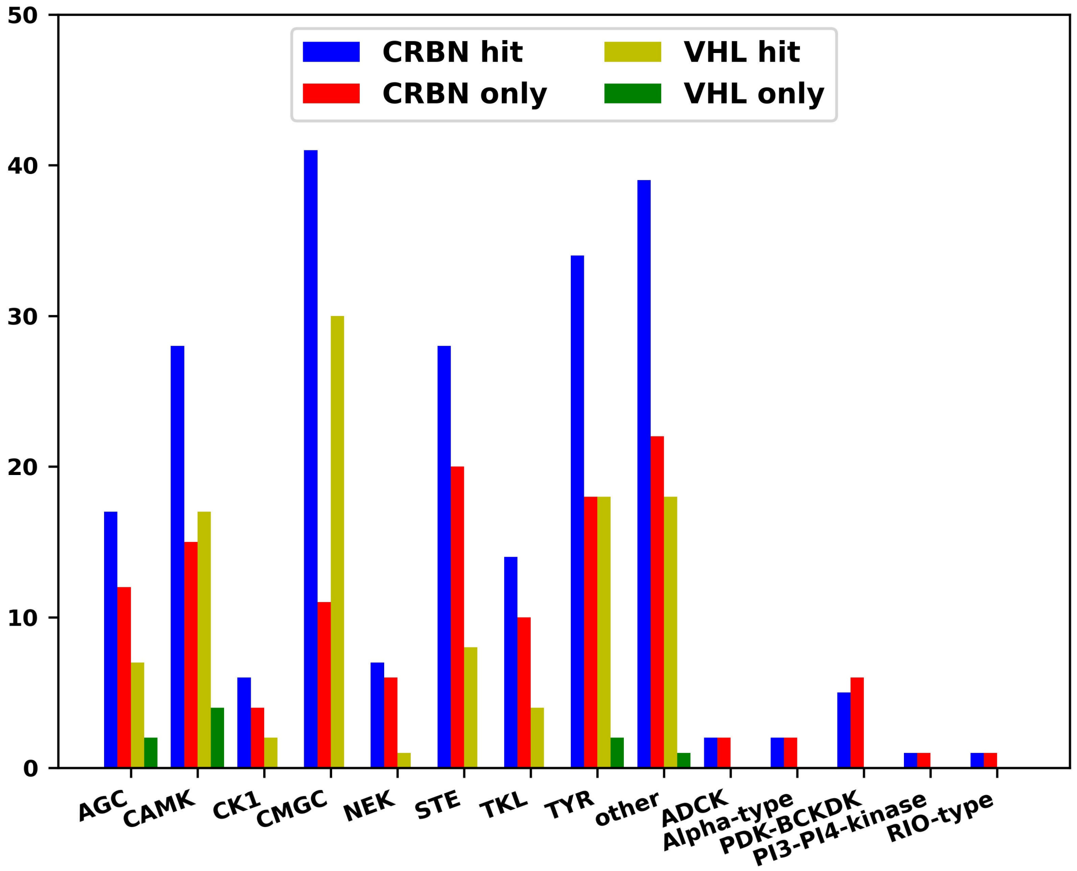

Fig Z. Distributions of CRBN induced degradations in different protein kinase families.

References

[1] Camacho C, Coulouris G, Avagyan V, Ma N, Papadopoulos J, Bealer K, et al. BLAST+: architecture and applications. BMC bioinformatics. 2009;10:1–9.
